# Challenges in Synthesis and Analysis of Asymmetrically Grafted Cellulose Nanocrystals via Atom Transfer Radical Polymerization

**DOI:** 10.1021/acs.biomac.1c00392

**Published:** 2021-06-01

**Authors:** Gwendoline Delepierre, Katja Heise, Kiia Malinen, Tetyana Koso, Leena Pitkänen, Emily D. Cranston, Ilkka Kilpeläinen, Mauri A. Kostiainen, Eero Kontturi, Christoph Weder, Justin O. Zoppe, Alistair W. T. King

**Affiliations:** ‡ Adolphe Merkle Institute, University of Fribourg, Chemin des Verdiers 4, 1700 Fribourg, Switzerland; § Department of Bioproducts and Biosystems, Aalto University, P.O. Box 16300, FI-00076, Aalto, Espoo Finland; ○ Department of Materials Science and Engineering, Universitat Politècnica de Catalunya, Av. Eduard Maristany 16, 08019 Barcelona, Spain; ⊥ Department of Wood Science, The University of British Columbia, 2424 Main Mall, Vancouver, British Columbia V6 T 1Z4, Canada; # Department of Chemical and Biological Engineering, The University of British Columbia, 2360 East Mall, Vancouver, British Columbia V6 T 1Z4, Canada; ∥ Materials Chemistry Division, Chemistry Department, University of Helsinki, A.I. Virtasen aukio 1, FI-00560 Helsinki, Finland

## Abstract

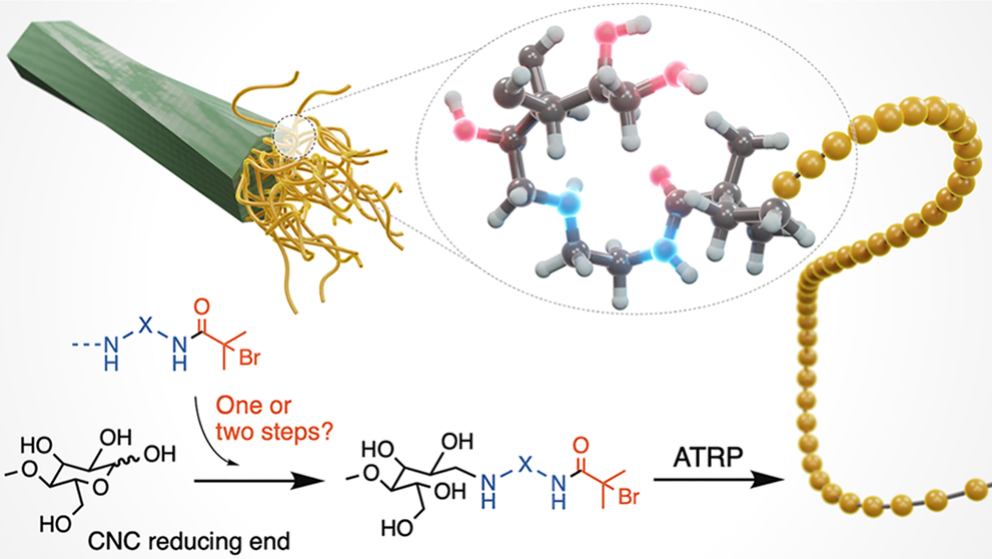

When cellulose nanocrystals (CNCs) are isolated from cellulose microfibrils, the parallel arrangement of the cellulose chains in the crystalline domains is retained so that all reducing end-groups (REGs) point to one crystallite end. This permits the selective chemical modification of one end of the CNCs. In this study, two reaction pathways are compared to selectively attach atom-transfer radical polymerization (ATRP) initiators to the REGs of CNCs, using reductive amination. This modification further enabled the site-specific grafting of the anionic polyelectrolyte poly(sodium 4-styrenesulfonate) (PSS) from the CNCs. Different analytical methods, including colorimetry and solution-state NMR analysis, were combined to confirm the REG-modification with ATRP-initiators and PSS. The achieved grafting yield was low due to either a limited conversion of the CNC REGs or side reactions on the polymerization initiator during the reductive amination. The end-tethered CNCs were easy to redisperse in water after freeze-drying, and the shear birefringence of colloidal suspensions is maintained after this process.

## Introduction

Cellulose nanocrystals (CNCs) are highly crystalline, rod-like nanoparticles with an anisotropic shape. CNCs are isolated in top-down processes from natural cellulose sources, such as wood and cotton, most commonly via a sulfuric acid-catalyzed hydrolysis.^1^ The acid hydrolyzes the dislocated regions of the macroscopic cellulose fibers, while the crystalline domains stay intact. The additional partial substitution of the CNC surface with sulfate half-ester groups (−OSO_3_^–^) results in charged particles that display a remarkable colloidal stability in water.^2,3^ Owing to their interesting physicochemical properties, CNCs are highly attractive for biobased or biocompatible applications in fields ranging from packaging to biomedical devices, cosmetics, nanocomposites and rheological modifiers in industrial liquids.^4−7^

The physicochemical properties of CNCs can be substantially influenced by functionalizing their surface hydroxy groups.^8−10^ Otherwise immiscible in hydrophobic matrices and nonpolar solvents, CNCs can be rendered more compatible by, for instance, surface-grafting of polymer chains, that are tailored toward the respective system.^10,11^ Consequently, this approach imparts the physicochemical properties of the grafted polymer to the CNC surface and further creates building blocks for functional colloidal dispersions and solid CNC-based materials.

Instead of modifying the surface of CNCs in a random manner, it is also possible to direct chemical modifications toward their ends.^12,13^ Selective end-group modification exploits the reducing end of cellulose and the parallel arrangement of the chains in the native cellulose I crystal,^12,14,15^ which dictates that all reducing end-groups (REGs) are localized at the same end of the CNCs. In contrast to uniform surface functionalization of hydroxy groups, the REG-modification preserves the unique surface chemistry and anisotropic character of the CNCs. In principle, polymer grafting via REG modification of CNCs enables the construction of Janus-type nanorods, which are otherwise unprecedented within the realm of biologically derived nanoparticles. A routine method to produce such structures would have a significant impact on the present scene of anisotropic nanoparticles whose applications are particularly strong in the biomedical field.^16−19^ However, precise knowledge of the chemistry and reliable characterization are mandatory before REG-modified CNCs can fulfill their promise. Therefore, this study focuses explicitly on the reaction mechanisms and analytics of CNCs with polymers grafted on their REGs, using advanced solution-state NMR-based analytics.^20,21^

Four different reaction schemes have been used previously to modify the end-groups of CNCs, including Knoevenagel condensation,^22^ reductive amination,^23^ Pinnick oxidation followed by amidation,^14,24−29^ and ligation.^15,30−33^ Nevertheless, little is known about how these reactions proceed when CNC REGs are involved, whose reactivity is governed by the tautomeric equilibrium known as mutarotation.^34^ As the equilibrium between the cyclic hemiacetals and the open chain aldehydes in CNC REGs is heavily biased toward the hemiacetals, the fraction of reactive (free) aldehydes is very low under aqueous conditions, where these species exist only as transient states.^12^ Therefore, the number of functionalities that can possibly be introduced at the chain ends, under realistic conditions, may be very small compared to the overall number of anhydroglucose units (AGUs) present in a CNC. This complicates the analysis of their modification using the standard analytical methods such as IR spectroscopy, solid-state NMR spectroscopy, and elemental analysis.^12^ So far, most reports quoting REG modification have relied on direct analysis using microscopy or on qualitative changes in the colloidal self-assembly characteristics, which cannot quantify the REG-modification and cannot rule out adsorption of reactants on the surface of the CNCs instead of genuine covalent modification. So far, only Heise et al.^21^ has provided direct spectroscopic evidence of REG modification.

CNCs with end-tethered polymer chains display very different characteristics than uniformly modified CNCs.^12^ Their self-assembly behavior is thought to be particularly useful to create ordered materials with defined structural hierarchies, including assemblies on surfaces and in nanocomposite materials. Two methods can be used to graft a polymer at the CNC REGs, that is, grafting *onto* and grafting *from*, although steric hindrance is often encountered while utilizing the first approach, whereas grafting from has, in general, the advantage of well-controlled polymer graft lengths and high polymer tether grafting density. However, reports on the grafting of polymers from the REGs of CNCs are limited.^26,27^ Surface-initiated atom transfer radical polymerization (SI-ATRP) is especially interesting in this regard due to its controllability, versatility, and tolerance to a variety of functional groups.^35^ Nevertheless, the bottleneck of site-selectively grafting from approaches involving CNC REGs remains the site-specific immobilization of ATRP-initiators on the REGs. Initial reports on the attachment of ATRP-initiators to the REGs of CNCs proceeded via a three-step pathway: (1) Pinnick oxidation (−CHO to – COOH), followed by (2) amidation (−COOH to −CONH–X–NH_2_), and (3) coupling of the ATRP-initiator via a *N*-hydroxysuccinimide (NHS) ester.^26,27^ However, after growing *N*-isopropylacrylamide and [2-(methacryloyloxy)ethyl] trimethylammonium chloride, a “patchy” distribution of polymer chains on the surface and the end-groups of the CNCs was observed, instead of a site-specific REG modification, presumably due to the presence of reactive carboxylic acid groups that remained as impurities on the surface of the CNCs, after hydrolysis.

To avoid this problem, we explored the use of reductive amination to selectively activate the REGs of CNCs and to introduce the ATRP-initiator. We studied two different pathways, including the direct reductive amination of the REGs with an amino-functionalized initiator, and a two-step pathway in which we first attached a diamine and then connected the terminal amines to the initiator moieties by NHS-mediated coupling. In order to tackle the challenging analysis of REG-modified CNCs, a combination of colorimetric methods with advanced solution-state NMR techniques was used to confirm the REG-modification. To assist in the NMR assignments, d-cellobiose served as a model cellulosic compound. Moreover, solution-state NMR spectroscopy was employed to confirm the REG grafting of sodium 4-vinylbenzenesulfonate (4-SS) as a model monomer for SI-ATRP from the modified REGs of CNCs. Finally, we studied how the site-selective polymer grafting affected the physicochemical properties of the CNCs.

## Experimental Section

### Materials

Sulfuric acid (97%), cotton Whatman ashless filter aid, sodium hydroxide, d-(+)-cellobiose, sodium 4-vinylbenzyl sulfonate (4-SS; ≥90%), 2,2′-bipyridine (bpy; ≥99%), sodium chloride, copper(II) bromide (99%), copper(I) bromide (99%), 2-picolineborane (2-PCB; 95%), α-bromoisobutyryl bromide (BiBB; 98%), 2-bromoisobutanoic acid (98%), *N*-hydroxysuccinimide ester (NHS-BiBB; 98%), 2,2′-(ethylenedioxy)-bis(ethylamine) (EBEA; 98%), ethyl α-bromoisobutyrate (EBiB; 98%), *N*-Boc-ethylenediamine (≥98%), *N*-Boc-2,2′-(ethylenedioxy)diethylamine (≥95%), triethylamine (for synthesis), poly-l-lysine (0.1% aqueous solution), ninhydrin reagent (2% in DMSO and lithium acetate buffer), methanol, ethanol, Dowex Marathon C hydrogen form (SAC exchange resin), AmberLite IRN-150 H^+^/OH^–^ ion-exchange resin, dialysis tubing cellulose membrane (MWCO 14 kDa), and thin-layer chromatography (TLC) silica gel 60 F_254_ sheets (20 × 20 cm, aluminum support) were all purchased from Sigma-Aldrich. Trifluoroacetic acid (TFA, M-Clarity quality level) was received from VWR. Millipore-processed deionized (DI) water was used with a resistivity of 18.2 MΩ·cm and will be referred to as ultrapure water. The NMR electrolyte solution containing tetrabutylphosphonium acetate ([P_4444_][OAc]) and DMSO-*d*_6_ (20:80 wt %) for CNC analysis was prepared as described previously.^20^

### Acid Hydrolysis

Cellulose nanocrystals were isolated by acid hydrolysis from cotton Whatman ashless filter aid (30 g) with sulfuric acid (64%, 420 mL) at 55 °C using a previously published protocol adapted from Frka-Petesic et al.^36,37^ The filter paper was cut into small pieces (ca. 1 × 1 cm) prior to blending with a Magic Bullet blender and added to the stirred acid at 55 °C. The mixture was stirred with a mechanical stirring rod for 35 min. The reaction was quenched by adding the sulfuric acid mixture to ice-cold ultrapure water (840 mL), followed by three subsequent centrifugations (20000 g, 30 min) and redispersion steps of the obtained pellet in DI water until the CNCs no longer sedimented. The CNCs were transferred to prewashed dialysis tubes (MWCO 14 kDa) and dialyzed against ultrapure water until the pH and conductivity remained constant. The aqueous suspension of the dialyzed CNCs (∼1 wt %) was then sonicated during 1 min, using a Branson Sonifier SFX550 (Thomas Scientific, Swedesboro, NJ, U.S.A.) in batches of 20 mL in an ice bath at 55% amplitude. Then, the CNCs were filtered through a Whatman glass microfiber filter (Grade GF/A, with pore size of 1.6 μm) and finally stored in acid form in the refrigerator. The unmodified CNC starting material had a length of 127 ± 49 nm, a height of 7 ± 2 nm, an aspect ratio of 19 ± 9, a sulfate half-ester group content of 235 ± 3 μmol −OSO_3_^–^/g CNC and a critical concentration for chiral nematic phase formation of 8 wt % (respectively, shown in Supporting Information, Figures S1–S3).

### Synthesis of ATRP-Initiators-1 and ATRP-Initiator-2

The ATRP-initiators *N*-(2-aminoethyl)-bromoisobutyrylamide (ATRP-initiator-1) and *N*-(2-(2-(2-aminoethoxy)ethoxy)ethyl)-bromoisobutyrylamide (ATRP-initiator-2; Supporting Information, Figure S4a) were synthesized as described by Houga et al. with several modifications.^38^ For a typical reaction, 0.2311 g (1.05 equiv) of Et_3_N and 1.05 equiv of Boc-protected diamine (*N*-Boc-ethylenediamine or *N*-Boc-2,2′-(ethylene-dioxy)diethylamine) were dissolved under continuous stirring in 5 mL of THF in a 25 mL round-bottom flask that was cooled in an ice bath. A solution of 0.5 g (1 equiv) of BiBB in 2 mL of THF was introduced dropwise with a syringe. After the addition of BiBB was complete, the ice bath was removed and the mixture was stirred for 48 h at room temperature. The Boc-protected intermediate was isolated by removing the salt precipitate by filtration through a glass fiber filter (G4). The filtrate was transferred into a 100 mL round-bottom flask and the solvent was removed in vacuo. The solid residue in the flask was then dissolved in 3 mL dichloromethane (DCM) and 1.5 mL of TFA were added dropwise (syringe) under continuous stirring and ice-cooling. After the addition was complete, the ice bath was removed and the mixture was stirred for 24 h at RT. The excess of TFA and solvent were then removed in vacuo and a repeated addition of DCM (5 times ca. 10 mL) ensured the complete removal of the excess of TFA. The solid residue was then dissolved in 10 mL DI water and extracted with DCM (5 × 5 mL). The water fraction was collected and reduced in vacuo, and the solid residue was dissolved in 5 mL of DCM/MeOH (70:30, v/v) and purified over a SiO_2_ flash column using DCM/MeOH (70:30, v/v) as mobile phase. ATRP initiator-1 and -2 were obtained after removing the DCM/MeOH mixture in vacuo and vacuum-drying. ATRP-initiator-1: ^1^H NMR (600 MHz, DMSO-*d*_6_) δ 3.37 (m, 2H), 2.92 (t, *J* = 6.2 Hz, 2H), 1.90 (s, 6H). HRMS (ESI) *m*/*z* calculated for C_6_H_13_BrN_2_O_1_ [M + H]^+^, 209.0284; found, 209.0285. *R*_f_ (by TLC on SiO_2_, mobile phase: 70:30 (v/v) DCM/MeOH): 0.54. ATRP-initiator-2: ^1^H NMR (600 MHz, DMSO-*d*_6_): δ 3.62 (t, *J* = 5.4 Hz, 2H), 3.58 (s, 4H), 3.49 (t, *J* = 6.2 Hz, 2H), 3.28 (m, 2H), 2.98 (q, *J* = 5.4 Hz, 2H), 1.88 (s, 6H). HRMS (ESI) *m*/*z* calculated for C_10_H_21_BrN_2_O_3_ [M + Na]^+^, 319.0628; found, 319.0629. *R*_f_ (TLC on SiO_2_, mobile phase: 70:30 (v/v) DCM/MeOH): 0.58.

### Reductive Amination on CNC Reducing Ends (CNC-RE-*g*-BiBB-1 and CNC-RE-*g*-NH_2_)

To a 250 mL round-bottom flask, 100 mL of an aqueous 1 wt % CNC suspension was added and the mixture was stirred with a magnetic stir bar, before ATRP-initiator-1 (0.1 g, 50 equiv) or 2,2′-(ethylenedioxy)bis(ethylamine) (0.127 mL, 50 equiv) were added to the reaction mixture. The equivalences were calculated based on the concentration of “aldehyde” (hemiacetal or aldehyde) groups, as quantified by the bicinchoninic acid (BCA) assay (*vide infra*). The pH of the reaction mixture was adjusted to 4.5 by adding acetic acid. The reaction mixture was heated to 70 °C, before 2-picoline-borane (2-PCB; 0.19 g, 100 equiv) was added to the reaction mixture. The reaction was left to stir for 3 days and every 24 h, 50 equiv of 2,2′-(ethylenedioxy)bis(ethylamine) (0.127 mL) or ATRP-initiator-1 (0.1 g), and 100 equiv of (2-PCB; 0.19 g) were added to the reaction mixture. After 3 days, the reaction mixture was cooled to RT and dialyzed against an aqueous NaOH solution at a pH of 9.5. After three exchanges of the aqueous NaOH solution, the modified CNCs were dialyzed against ultrapure water until the pH of the dialysis water remained constant, the CNCs were then washed in a stirred cell with ultrapure water and ethanol in order to remove the potentially adsorbed species from the surface of the CNCs. The modified CNC suspensions were stored at a concentration of about 1 wt % in the refrigerator at 4 °C. This reaction was also performed on the REG-oxidized CNC-RE-COOH with the addition of 2,2′-(ethylenedioxy)bis(ethylamine) in order to quantify the adsorption on the surface of the CNCs and on unreacted CNCs without the addition of amine in order to determine the effect of 2-PCB on the REGs. The modified CNC suspensions were stored at a concentration of about 1 wt % in the refrigerator at 4 °C.

### ATRP-Initiator Attachment via NHS-Mediated Coupling (CNC-RE-*g*-BiBB-2)

An aqueous dispersion of amine-functionalized CNCs (100 mL, 1 wt %) was added to a 250 mL round-bottom flask containing 0.14 g of Na_2_HPO_4_ (10 mM), while stirring with a magnetic stir bar. The pH of the mixture was adjusted to 7.2 with a dilute solution of NaOH and the mixture was sonicated for 10 min. In a separate vial, 87 mg of NHS-BiBB were dissolved in 5 mL of DMSO and the solution was added dropwise (syringe) to the flask containing the aqueous dispersion of amine-functionalized CNCs. The reaction was left to react for 4 h at room temperature prior to performing dialysis against ultrapure water until the conductivity of the wash water was stable. The modified CNC suspension was stored in the refrigerator at 4 °C.

### Grafting Polystyrenesulfonate (PSS) from CNC Reducing Ends (CNC-RE-*g*-PSS-1 and CNC-RE-*g*-2)

To a 250 mL Schlenk flask containing a magnetic stir bar, 5.4 mg of CuBr_2_, 0.19 g of 2,2′-bipyridyl, and 5 g of sodium 4-vinylbenzenesulfonate were added. Then, 25 mL of an aqueous suspension containing CNCs modified with the ATRP initiator (CNC-RE-*g*-BiBB-1orCNC-RE-*g*-BiBB-2) and 25 mL of methanol were added, and the reaction mixture was subjected to three freeze–pump–thaw cycles and refilled with N_2_ gas. During the fourth freeze–pump–thaw cycle, 50 mg of Cu(I)Br was added under a nitrogen atmosphere to the Schlenk flask containing the frozen CNCs. The Schlenk flask was then closed and an additional vacuum and refill cycle was performed. The color of the reaction turned dark brown after the addition of Cu(I)Br and thawing of the suspension. The reaction mixture was then stirred at room temperature under a N_2_ atmosphere for 18 h. The reaction was stopped by opening up the flask to air. The mixture was transferred to centrifuge tubes and centrifuged for 15 min at 9000 rpm. A white pellet was formed, which was redispersed in ultrapure water and passed through a strong acid cation exchange column to remove residual copper, which also protonates the CNC-RE-*g*-PSS. The modified CNCs were then dialyzed against ultrapure water until the conductivity of the wash-water was constant. Half of the suspension was freeze-dried after neutralizing the suspension with 0.1 M NaOH, and the other half was kept in the acid form obtained after dialysis at a concentration of about 0.4 wt % in the refrigerator at 4 °C.

### Oxidation of CNC Reducing Ends (CNC-RE-COOH)

CNCs (100 mL, 1 wt %) were stirred in a 250 mL round-bottom flask. NaClO_2_ (2.83 g) was added to the CNC suspension. The reaction mixture was stirred for 30 min before the pH was adjusted to 3.5 with acetic acid. The mixture was stirred for 48 h at room temperature. The CNCs were subsequently dialyzed against ultrapure water until the pH of dialysis water was constant. The oxidized CNCs were kept at a concentration of 1 wt % in the refrigerator at 4 °C.

### Bromoisobutyryl-Cellobiosylamide: One-Step Pathway (M1)

In a 25 mL round-bottom flask, 0.1 g of d-(+)-cellobiose (0.29 mmol, 1 equiv) was dissolved in 3 mL of DI water. To this solution, 0.14 g of NH_2_-terminated ATRP-initiator-1 (0.44 mmol, 1.5 equiv), dissolved in 2 mL of DI water, was added (solution pH = 3.7). Then, 2-PCB (0.09 g, 0.88 mmol, 6 equiv) was added with another 1 mL of DI water. The reaction mixture was heated to 70 °C for 72 h under constant stirring. After completion, the high excess of borane reducing agent was removed by an acid-MeOH treatment. Thus, 1.2 mL of TFA (corresponding to a TFA concentration of 20 vol % in the reaction mixture) was added slowly with a syringe, and the mixture was stirred at RT for 1 h. Afterward, TFA and water were removed in vacuo and water was added repeatedly (ca. 5 × 10 mL) for the thorough removal of all acid residues. Then, the viscous residue was dissolved in 20 mL of MeOH and stirred for 30 min at 45 °C. The MeOH was removed in vacuo with repeated (5 × 5 mL) MeOH addition and removal in vacuo. Then, the viscous residue was dissolved in 20 mL of water and extracted (5 × 5 mL) ethyl acetate. The water phase was collected and reduced in vacuo and the product was obtained by precipitation from 0.5 mL 1:1 MeOH/water (v/v) into ice-chilled acetone. ^1^H and ^13^C NMR assignments were performed (Supporting Information, Figure S5). The sample was also found to contain small amounts of starting d-(+)-cellobiose and was partially hydrolyzed from the BiBB-Br to BiBB-OH. HRMS (ESI) *m*/*z* calculated for C_18_H_35_BrN_2_O_11_ [M + H]^+^, 535.1497; found, 535.1493 (Supporting Information, Figure S6).

### Cellobiosylamine: Two-Step Pathway (M2)

*N*-2,2′-(Ethylenedioxy)diethylamine-cellobiosylamine M2 was synthesized under similar conditions as model compound M1. To a solution of 1 mmol d-(+)-cellobiose (0.3423 g, 1 equiv) in 5 mLof DI water (25 mL round-bottom flask), 1.5 mmol 2,2′-(ethylenedioxy)bis(ethylamine) (0.2223 g), dissolved in 1 mL of DI water, was added and the pH was adjusted to 4.5 with glacial acetic acid. Then, 2-PCB (3 mmol, 0.3209 g) was added with another 1 mL of DI water. The reaction mixture was heated to 70 °C for 24 h under constant stirring. After the reaction, the aqueous reaction mixture was extracted with ethyl acetate (5 × 5 mL). The water phase was collected and reduced in vacuo. Afterward, the viscous residue was dissolved in 1.5 mL of water/MeOH (50:50, v/v) and precipitated into ice-chilled acetone. Further purification (e.g., for removal of residual borane compounds) was achieved by preparative-scale liquid chromatography, as described in the Supporting Information (Section S2c). Two fractions were collected from 58 injections after 3.80–4.30 min for fraction 1 and 4.30–4.65 min for fraction 2. The solvent from the combined fraction 1 was removed in vacuo and the isolated cellobiosylamine M2 was subjected to NMR and mass analysis. ^1^H and ^13^C NMR assignments were performed (Figure S7). HRMS (ESI) *m*/*z* calculated for C_18_H_38_BrN_2_O_12_ [M + Na]^+^, 645.1841; found, 645.1833 (Figure S8).

### Bromoisobutyryl-Cellobiosylamide: Two-Step Pathway (M3)

For the synthesis of model compound M3, 0.1 g cellobiosylamine M2 (0.21 mmol, 1 equiv) was charged to a 25 mL round-bottom flask and dissolved in 10 mL of 10 mM Na_2_HPO_4_ solution. The pH was adjusted to 7.2 with acetic acid. A total of 0.084 g of NHS-BiBB (0.32 mmol, 1.5 equiv) was dissolved in 1 mL of DMSO, and the solution was added portionwise with a syringe to the reaction mixture under ice-cooling. After the addition was complete, the ice bath was removed and the reaction mixture was stirred at RT for 4 h. The aqueous solution was then rinsed through a flash column filled with H^+^/OH^–^ ion-exchange resin (AmberLite IRN-150) followed by (5 × 6 mL) extractions with ethyl acetate and reduction of the water fraction in vacuo. The viscous residue was then dissolved in 1 mL of 1:1 (v/v) MeOH/water, precipitated into ice-chilled acetone and the fine precipitate particles were isolated by centrifugation followed by vacuum-drying for removing residual solvent. The product contained significant amounts of the starting cellobiosylamine, thus the product yield was low. The main NMR resonances are assigned in the Supporting Information (Figure S9). Mass spectrometry analysis confirmed the presence of the product: HRMS (ESI) *m*/*z* calculated for C_22_H_43_BrN_2_O_13_ [M], 622.1949; found (5 ppm range), 645.1833 (M + Na)^+^ (Figure S10).

### PSS Homopolymer

To a 100 mL Schlenk flask containing a magnetic stir bar, 5.4 mg of CuBr_2_, 0.19 g of 2,2′-bipyridyl, and 2.95 g of sodium 4-vinylbenzenesulfonate were added. Then, 10 μL of ethyl α-bromoisobutyrate and 30 mL of a 1:1 methanol–water mixture was added, and the reaction mixture was subjected to three freeze–pump–thaw cycles and refilled with N_2_ gas. During the fourth freeze–pump–thaw cycle, 50 mg of Cu(I)Br was added under a nitrogen atmosphere to the Schlenk flask containing the frozen solution. The Schlenk flask was then closed and an additional vacuum cycle was performed before refilling the flask with N_2_ gas. The reaction was then stirred at room temperature under a N_2_ atmosphere for 24 h. The polymer was then dialyzed (MWCO 1 kDa) against DI water until the conductivity of the wash water remained constant. The obtained polymer was then stirred with a silica Cu scavenger to remove the Cu from the mixture, followed by a filtration step through a fritted glass filter to remove the silica from the solution. The polymer had a number-average molecular weight (*M*_n_) = 32000 g mol^–1^, *Đ* = 1.4. ^1^H and ^13^C NMR assignments were performed and are shown in Figure 7.

### Reducing End-Group Quantification

Bicinchoninic acid assay (BCA) was used to quantify the number of reducing end-groups on the CNCs as previously reported by Risteen et al.^27^ Step 1: two solutions were prepared: Solution A (pH 9.7) contained Na_2_CO_3_ (5.43 g), NaHCO_3_ (2.42 g), and BCA disodium salt hydrate (0.194 g) in ultrapure water (100 mL); Solution B contained CuSO_4_·5H_2_O (0.125 g) and l-serine (0.126 g) in ultrapure water (100 mL). Step 2: a calibration curve was prepared with a 0–80 × 10^–6^ M glucose solution. For each concentration in the calibration curve, 2 mL of glucose solution, 1 mL of solution A, and 1 mL of solution B were added to a glass vial with a cap and a stirring bar. Step 3: to the glass vials containing 4 mL of the aqueous mixture, modified CNCs (5.0 × 10^–3^ g) were added together with 1 mL of solutions A and B. All the vials (CNC suspensions and glucose solutions) were heated to 75 °C for 30 min in a water bath. The vials were then cooled to room temperature, the CNC samples were centrifuged at 5000 rpm for 10 min to remove the CNCs, and the absorbance of the glucose solutions and the supernatants of the CNC samples were measured at 560 nm. The experiments were done in triplicate.

### Ninhydrin Assay

The procedure was performed as reported by Risteen et al.^27^ A calibration curve was obtained using 2-ethyl-1-hexylamine in DI water with concentrations ranging from 1 × 10^–5^ to 2.5 × 10^–4^ M. To a microwave vial, 2 mL of the 2-ethyl-1-hexylamine and 1 mL of the ninhydrin reagent solution (ninhydrin and hydrindantin with lithium acetate buffer, pH 5.2) were added and sealed under N_2_, shaken by hand and heated for 30 min at 100 °C. The tubes were then cooled down to room temperature and 5 mL of 50% (v/v) ethanol/water was added to the mixture. A total of 15 s of stirring using a Vortex mixer was performed in order to oxidize the excess of hydrindantin in the solution. The absorbance was recorded at 570 nm. The same procedure was followed for the CNC samples except that after vortex mixing, the CNCs were removed from the suspension by centrifugation (5000 rpm, 10 min). The absorbance of the supernatants was measured at 570 nm. The experiments were done in triplicate.

### Solution-State NMR Characterization

In a typical sample dissolution procedure, 50 mg of dry cellulosic material was introduced into 950 mg of [P_4444_][OAc]/DMSO-*d*_6_ stock electrolyte, in 4 mL sealed vials, equipped with stirring bars. The overall sample volume changed from sample to sample, but the concentration was kept at ∼5 wt % for all. Model compounds and the PSS homopolymer sample were dissolved to give typically 1–8 wt % solutions in DMSO-*d*_6_. These were initially stirred at RT to see if the samples dissolved. If not, they were heated at 65 °C under inert atmosphere. Once the solutions were clear and visually homogeneous, the samples were transferred into 5 mm NMR tubes (Wilmad-Labglass Co., U.S.A.) for analysis. Spectra were recorded using a Bruker Avance 600 MHz NEO spectrometer. The experiments were recorded using an inverse triple resonance probe-head (^1^H/^19^F, ^13^C, ^31^P). Standard ^1^H 1D experiments were recorded for all samples. Diffusion-edited 1D ^1^H experiments were measured for all cellulose samples using a 1D bipolar-pulse pair stimulated echo (BPPSTE) pulse sequence (“ledbpgp2s1d” in the Bruker TopSpin 4.0 pulse program library). Multiplicity-edited Heteronuclear Single Quantum Correlation (HSQC)^39^ experiments (“hsqcedetgpsisp2.2” in the Bruker TopSpin 4.0 pulse program library) were recorded for all samples. 2D HSQC-total correlation spectroscopy (HSQC-TOCSY)^40^ experiments (“hsqcdietgpsisp.2” in the Bruker TopSpin 4.0 pulse program library), with short (15 ms) TOCSY mixing times, were recorded for some samples to aid in resonance assignment. Heteronuclear multiple bond correlation (HMBC)^39^ experiments (“hmbcgplpndqf” in the Bruker TopSpin 4.0 pulse program library) were recorded for some samples, also to aid in signal assignment. All NMR measurements were conducted at a sample temperature of 65 °C. Chemical shifts in ^1^H and ^13^C ppm scales were calibrated against the DMSO-*d*_6_ signals (2.50 ppm for residual ^1^H and 39.52 ppm for ^13^C). All spectra were processed using Bruker TopSpin 4.0.6 and MestReNova 10.0.2^41^ software. Further 1D data processing was completed using Fityk 1.3.1.^42^ Full NMR experimental and conditions are given in the Supporting Information (Section S4a).

### Mass Spectrometry

The LC-MS analysis was performed on an Agilent 6350 QTOF mass spectrometer connected to an Agilent 1260 HPLC system (Agilent Technologies). The injection was performed using an injection volume of 2 μL with a flow rate of 0.250 mL/min (50:50 acetonitrile/ultrapure water). The ionization was done using a dual electrospray in positive ion mode. The instrumental parameters were set as follows: capillary voltage, 3500 V; source temperature, 300 °C; drying gas, 11 L/min; nebulizer pressure, 25 psi; fragmentor and skimmer voltages, 150 and 65 V, respectively. Data was acquired (2 Hz) in profile mode using the software MassHunterWorkstation (Agilent Technologies). The spectra were acquired over a mass-to-charge (*m*/*z*) range of 120–1100. Reference mass correction on each sample was performed with a continuous infusion of purine (*m*/*z* 121.0509) and hexakis(1*H*,1*H*,3*H*-tetrafluoropropoxy) phosphazine (*m*/*z* 922.0098; Agilent Technologies). The mass accuracy of the instrument using external calibration was specified to be ≤3 ppm.

### Conductometric Titrations

Conductometric titrations were performed with a SevenCompact Duo pH/conductivity probe (Mettler Toledo) following the procedure by Beck et al.^43^ Prior to conductometric titration, the suspensions were passed through a strong acid cation exchange resin column (12 g SAC/1 g CNCs). A 10 mM NaOH solution was prepared, its concentration was standardized with the primary standard potassium hydrogen phthalate (KHP) by using phenolphthalein as an indicator. Then, 20 mL of a 0.5 wt % CNC suspension containing 1 mM NaCl was titrated against 10 mM NaOH solution. The first equivalence point indicates the number of sulfate half ester groups and the difference between the first and the second equivalence point indicates the number of carboxylic acid groups. The titrations were performed in triplicate and the average of the measurements is reported with its standard deviation.

### Atomic Force Microscopy (AFM)

Mica was freshly cleaved with tape prior to being functionalized with an aqueous solution of poly-l-lysine (0.01%) by drop-casting (40 μL) of the solution. After 30 s, the poly-l-lysine was washed off with ultrapure water and dried under a nitrogen flow. Aqueous CNCs suspensions (0.001 wt %, 40 μL) were then drop casted onto the functionalized mica surface, after 30 s, the surface was washed with ultrapure water, and dried under a nitrogen flow. The images were acquired with a Park NX10 microscope, in tapping mode with TAP300Al-G probes at room temperature. Images were recorded with 4096 × 4096 pixels of resolution in a 25 × 25 μm and were subsequently cropped in Illustrator to have a final image of 5 × 5 μm. The CNC size and cross-section were measured using the XEI software (Park Systems) on more than 1000 rods. The aspect ratio of the CNCs was calculated by dividing the length of the CNCs by their height, and aspect ratio’s lower than 10 were removed manually, as these are impurities that do not fit the definition of CNCs.^44^ The reported confidence intervals are the standard deviation of the average particle dimensions.

### ζ-Potential and Dynamic Light Scattering (DLS)

A suspension of 0.25 wt % (unmodified and modified) CNCs containing 5 mM NaCl was prepared to measure the ζ-potential. Prior to measurement, the suspension was bath-sonicated for 10 min in ice-cold water. The measurements were recorded above the p*K*_a_ of the sulfate groups on the CNCs at a pH of 3.17, 3.24, and 3.48 for unmodified CNCs, CNC-RE-*g*-PSS-1, and CNC-RE-*g*-PSS-2, respectively. The measurements were recorded on a Malvern Panalytical Zetasizer Nano-ZS. A total of 10 measurements were taken, and the average was reported with its standard deviation. The ζ-potential suspension was diluted 10 times for the DLS “apparent size” measurements, resonicated, and measured on the same instrument. We use the terminology “apparent size” in recognition of the limitation of DLS that assumes spherical particles, which is not the case for rod-shaped CNCs, but we believe the relative size comparison and inference of suspension quality are useful nonetheless.

### Elemental Analysis

Elemental analysis (C, H, N, S (%)) was performed on freeze-dried CNC samples by the Microelemental Analysis Laboratory of ETH Zürich. The C, H, and N content was quantified on a TruSpec Micro (LECO). The combustion products were analyzed by infrared spectroscopy. The S content was determined with an EuroVector (HEKAtech) elemental analyzer. All results are given in mass%.

### Fourier-Transform Infrared Spectroscopy (FTIR)

Fourier-transform infrared spectroscopy (FTIR) spectra were collected on a PerkinElmer Spectrum 65 spectrometer in attenuated total reflectance (ATR) mode (Universal ATR model, 1.66 μm depth of penetration). Freeze-dried CNC samples were compacted inside a syringe to make a small pellet prior to analysis. A total of eight scans at a 4 cm^–1^ resolution and a wavenumber range of 4000–600 cm^–1^ were acquired per sample. The spectra were plotted in Origin, and the peak intensity at 1180 cm^–1^ (corresponding to the antisymmetric vibrational adsorption peaks of the sulfonate group^45^) was compared to the intensity at 1424 cm^–1^ (corresponding to the CH_2_ scissor motion in cellulose^46^), and the ratio of the two peaks was calculated by dividing the intensity of the peak corresponding to the PSS by the intensity of the peak corresponding to cellulose.

### Liquid Crystalline (LC) Phase Separation

CNC suspensions were concentrated by slow evaporation of water at RT while stirring with a magnetic stir bar. The concentrated CNC suspensions were diluted into vials using purified water and a NaCl solution (30 mM) to obtain the targeted concentration of CNCs ranging from 3.5 to 10 wt % with 1 mM NaCl, depending on the CNCs. The suspensions were added to small flat glass capillary tubes obtained from VitroTubes (0.6 × 0.3 × 5 mm) and left to self-assemble/phase separate.^47,48^ The suspensions in the capillaries were equilibrated for 7 days prior to taking the photographs in between crossed polarizers.

### Polarized Optical Microscopy (POM)

POM images of the capillary tubes filled with CNC suspensions (as described above) were acquired in reflection mode on an Olympus BX51 microscope equipped with a digital camera. Polarized optical microscopy images were collected with crossed linear polarizers, which were oriented horizontally and vertically relative to the analyzer.

### Surface Tension Measurements

Pendant drop measurements of unmodified CNCs and CNC-RE-*g*-PSS-1 and CNC-RE-*g*-PSS-2 at 0.4 wt % were performed on a DataPhysics OCA instrument. The measurements were repeated five times, and the average is reported with its standard deviation.

## Results and Discussion

### Introduction of the ATRP-Initiator at the REGs of CNCs

Rod-shaped CNCs with an average length of 133 ± 62 nm, a height of 7 ± 3 nm (Figure S1), and a surface charge of 235 ± 3 μmol −OSO_3_^–^/g CNC (conductometric titration data, Figure S2) were isolated by acid hydrolysis from cotton filter aid. These CNCs served as the starting material for the synthesis of asymmetric CNC–polymer hybrids.

#### Route 1: One-Step Pathway

**Scheme 1:**
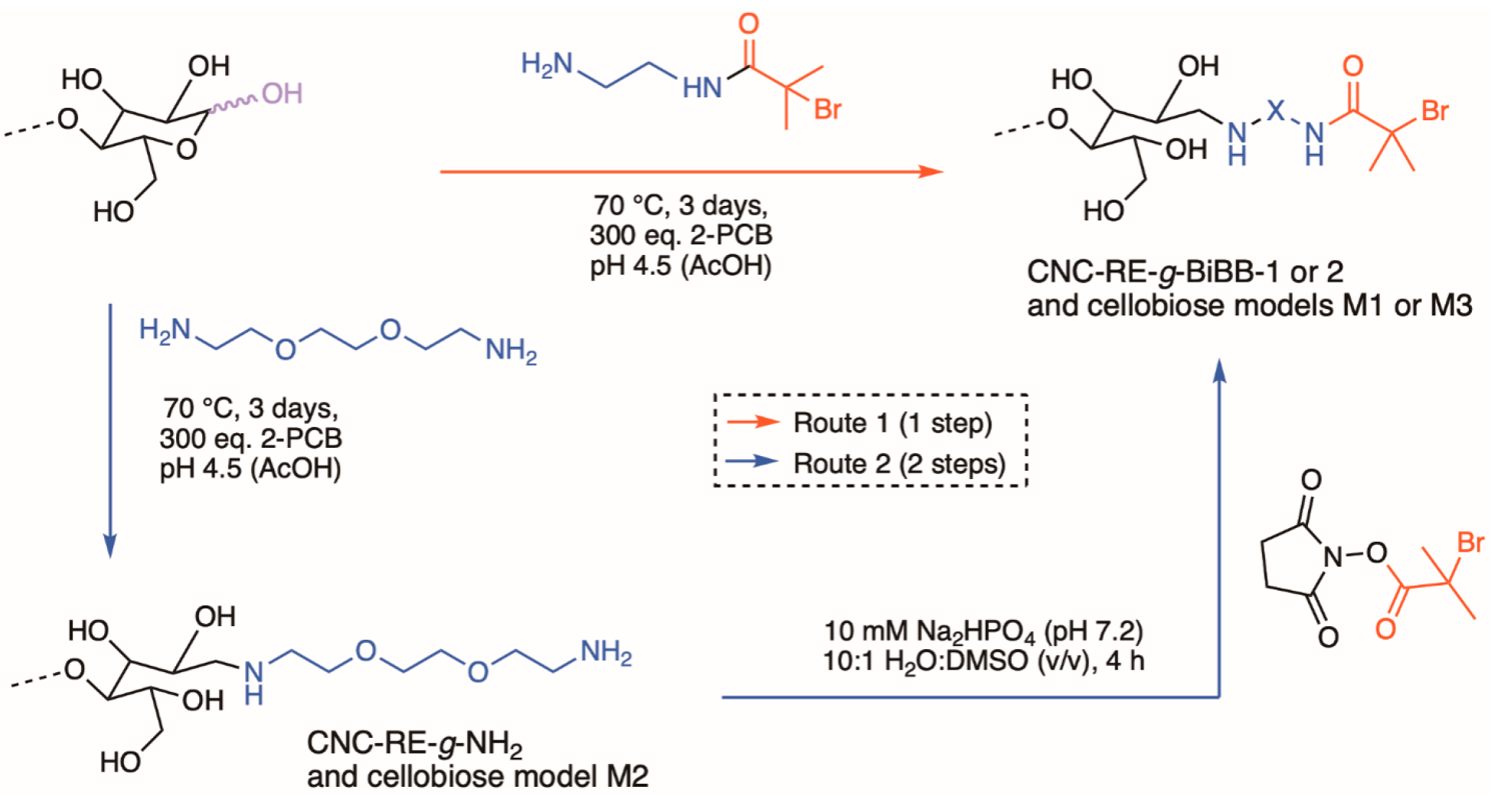
Scheme Showing the Two Routes That Were Used To Attach an ATRP-initiator to the REGs of CNCs The first route consists of attaching the presynthesized amine-terminated ATRP-initiator-1 to the REGs, forming compound CNC-RE-*g*-BiBB-1. The second route involves two steps: first, a diamine is attached to the CNC REGs, forming CNC-RE-*g*-NH_2_, before an ATRP-initiator is attached in the form of a NHS-activated ester, affording CNC-RE-*g*-BiBB-2.

As illustrated in Scheme 1, two different approaches were used to attach an ATRP-initiator selectively to the REGs of CNCs. The first reaction route leading to CNC-RE-*g*-BiBB-1 was based on a one-step pathway in which the amino-terminated ATRP-initiator-1 was linked to the REGs via a direct reductive amination in water, at 70 °C and acidic pH (4.5), using 2-picolineborane (2-PCB) as reductant. Concomitantly, we investigated the conversion of d-cellobiose following similar reaction conditions (model compound M1), which facilitated the solution-state NMR analysis of CNC-RE-*g*-BiBB-1.

Colorimetric methods were used to monitor the REG modification of the CNCs (Table 1). The bicinchoninic acid (BCA) assay showed that the concentration of aldehyde end-groups was reduced from 16 μmol CHO/g CNC for the unmodified CNCs to 7 μmol CHO/g CNC for CNC-RE-*g*-BiBB-1, suggesting a successful REG modification. Nevertheless, according to the BCA assay, about 44% of the end-groups remained unmodified, which might be explained by the limited availability of the REGs due to mutarotation,^34^ and due to the increased steric hindrance at the REGs as the modification proceeds. However, it must be pointed out that the end-group conversion through the BCA assay probes the concentration of available aldehyde groups at pH 10 (buffer pH) and not the total concentration of aldehyde groups.^37^ Additionally, unmodified CNCs were subjected to the reductive amination reaction conditions without the presence of the ATRP-initiator in order to exclude a possible side-reaction of the reductant 2-PCB with the CNC REGs (CNC + 2-PCB, Table 1). The BCA assay confirmed that the reducing agent had no effect on the REGs of the CNCs, as 16 μmol CHO/g CNC were quantified prior to and after the reaction.

**Table 1:** Concentrations of Aldehyde Reducing Ends and Primary Amine Groups in the Pristine CNCs and the Modified CNCs after Amine and ATRP-Initiator Attachment^a^

Reaction	Product	No. of reducing ends (μmol CHO/g CNC)	ninhydrin assay (μmol NH_2_/g CNC)
initial	CNC-CHO	16	2
route 1	CNC-RE-*g*-BiBB-1	7	n/a
route 2	CNC-RE-*g*-NH_2_	2	21
	CNC-RE-*g*-BiBB-2	2	5
blank reactions	CNC-RE-COOH	2	0
	mixture CNC-RE-COOH + NH_2_-X-NH_2_	1	4
	CNC + 2-PCB	16	3

a The oxidation of the REGs of the CNCs and the adsorption of the amine on the surface are also reported.

**Figure 1:**
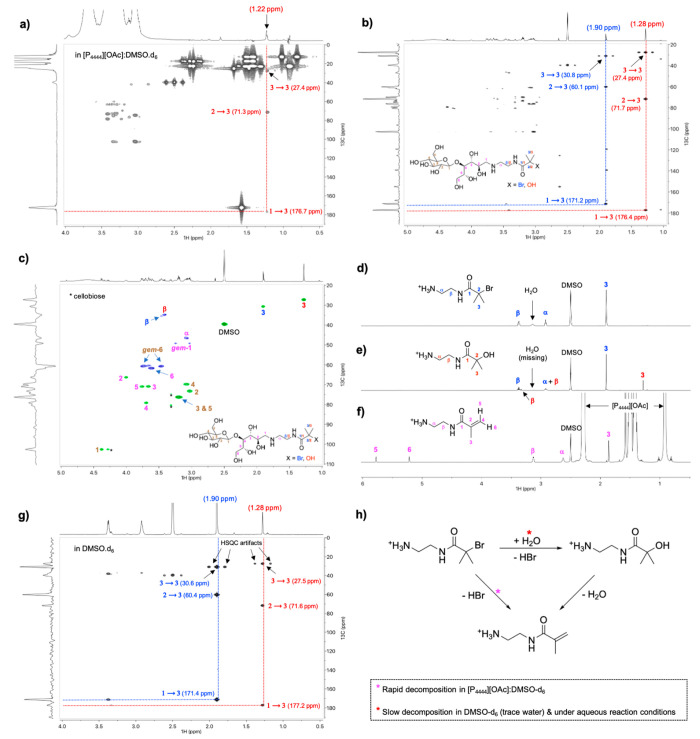
NMR spectra: (a) ^13^C HMBC spectrum of CNC-RE-*g*-BiBB-1 just after dissolution into [P_4444_][OAc]/DMSO-*d*_6_, (b) ^13^C HMBC spectrum of cellobiose model compound M1 (mixture of BiBB-Br and BiBB-OH from the reductive amination step) in DMSO-*d*_6_, (c) Multiplicity-edited HSQC spectrum of cellobiose model compound M1 in DMSO-*d*_6_, (d) ^1^H NMR spectrum just after dissolution of ATRP-initiator-1 in DMSO-*d*_6_, (e) ^1^H NMR spectrum of ATRP-initiator-1in DMSO-*d*_6_ (after heating for 24 h at 65 °C), (f) ^1^H NMR spectrum of ATRP-initiator-1 in [P_4444_][OAc]/DMSO-*d*_6_ (after heating for 5 min at 65 °C), (g) ^13^C HMBC spectrum of ATRP-initiator-1 in DMSO-*d*_6_ (after heating for 24 h at 65 °C), and (g) proposed decomposition mechanism for ATRP-initiator-1 in the NMR solvents or under reaction conditions.

The selective attachment of ATRP-initiator-1 to the CNC REGs was further confirmed by using an advanced solution-state NMR method for analyzing crystalline cellulose.^20,21^ This involved dissolution of freeze-dried CNCs into the [P_4444_][OAc]/DMSO-*d*_6_ electrolyte, prior to NMR analysis at 65 °C. Gratifyingly, the ^13^C HMBC NMR spectra of CNC-RE*-g*-BiBB-1 (Figure 1a) revealed the presence of a BiBB-like moiety, featuring signals at chemical shifts that are characteristic of a carbonyl group (177.2 ppm ^13^C chemical shift) and a quaternary carbon (71.6 ppm ^13^C chemical shift) that are correlated with aliphatic resonances (27.5 ppm ^13^C chemical shift and 1.22 ppm ^1^H chemical shift). These aliphatic resonances also have HMBC correlations to themselves, indicating that these equivalent alkyl resonances were attached through a covalent bond. The diffusion-edited ^1^H trace for the HMBC (Figure 1a, ^1^H trace) demonstrates that the aliphatic signal at 1.22 ppm is also slow-diffusing, thus, it is attached to the cellulose. The same 1D signals and 2D correlations are also clearly visible in the HMBC and multiplicity-edited HSQC spectra for the cellobiose model M1 (Figure 1b,c, at 65 °C in DMSO-*d*_6_), yet, with an additional BiBB-like spin system (with associated HMBC correlations at a ^1^H chemical shift of 1.90 ppm).

#### Side Reactions of the ATRP-Moieties

These proposed BiBB-like resonances (Figure 1a–c), as a consequence of the initiator attachment to the CNC or cellobiose REGs, were further assigned with the help of ATRP-initiator-1 (Figure 1d–g). Figure 1d shows the ^1^H NMR spectrum of ATRP-initiator-1 in DMSO-*d*_6_. However, the BiBB moiety CH_3_ groups appear at a ^1^H chemical shift of 1.90 ppm; downfield from those aliphatic signals that appeared in the CNC-RE-*g*-BiBB-1 HMBC ^1^H trace (Figure 1a). This observation suggests a decomposition reaction happening at the quaternary carbon of the BiBB moiety during the reductive amination, as has been observed in previous reports,^49^ and during the subsequent analysis. Possible side reactions on the ATRP-initiator-1 were elucidated in more detail by heating solutions of this compound under different conditions and subsequent NMR analysis.

When ATRP-initiator-1 was heated at 65 °C overnight (24 h) in DMSO-*d*_6_, a new singlet appeared at a ^1^H chemical shift of 1.28 ppm (Figure 1e), in addition to a signal at 1.90 ppm. This singlet also had an equivalent set of ^13^C HMBC correlations (Figure 1g), consistent with a BiBB-like spin system. The disappearance of the water signal suggested that the water may have been consumed in a hydrolysis reaction of BiBB-Br to BiBB-OH. However, the new alkyl signals at 1.26 ppm in the ^1^H spectrum were correlated (HMBC) with ^13^C resonances at 27.5, 71.6, and 177.2 ppm. These are consistent with literature values for 2-hydroxy-2-methylpropionamide in D_2_O,^50^ further confirming the hydrolysis of BiBB-Br to BiBB–OH. Upon heating a solution of ATRPinitiator-1 into the more basic [P_4444_][OAc]:DMSO-*d*_*6*_ NMR solvent (Figure 1f), signals corresponding to a methacrylamide product appeared, that is, a product that is produced either via water or HBr elimination from BiBB-OH or BiBB-Br, respectively. Thus, the BiBB-Br moiety is sensitive toward hydrolysis and elimination. Such reactions occur during NMR analysis and under the conditions required for the reductive amination (Figure 1h). During NMR analysis, conversion of BiBB-Br to methacrylamide was rather rapid in [P_4444_][OAc]/DMSO-*d*_6_ (methacrylamide was observed after 5 min at 65 °C), whereas the hydrolysis to BiBB-OH in DMSO-*d*_6_ and further elimination of BiBB-OH to methacrylamide, in [P_4444_][OAc]/DMSO-*d*_6_, were rather slow (partial conversion overnight at 65 °C). While these mechanisms occurred during NMR analysis, the hydrolysis reaction of BiBB-Br to BiBB-OH and potential elimination to methacrylamide was also expected to take place during the reductive amination reaction, when attaching the ATRP-initiator-1, at 70 °C in water to the REGs of the CNCs.^49^ This was further studied by heating a solution of ATRP-initiator-1 in D_2_O for 24 h at 70 °C, simulating the conditions during the reductive amination. Both OH-substitution and elimination to methacrylamide were observed after heating. Moreover, the mass spectrum of cellobiose model M1 (Supporting Information, Figure S6), acquired directly after the reductive amination and the compound workup, supports that at least some of the ATRP-initiator decomposition happened before NMR analysis. Both degradation products were clearly identified at *m*/*z* 473.23 (elimination product, [M + H]^+^) and 455.22 (hydrolysis product, [M + H]^+^). These factors are important when drawing conclusions at each reaction step.

#### Route 2: Two-Step Pathway

**Figure 2:**
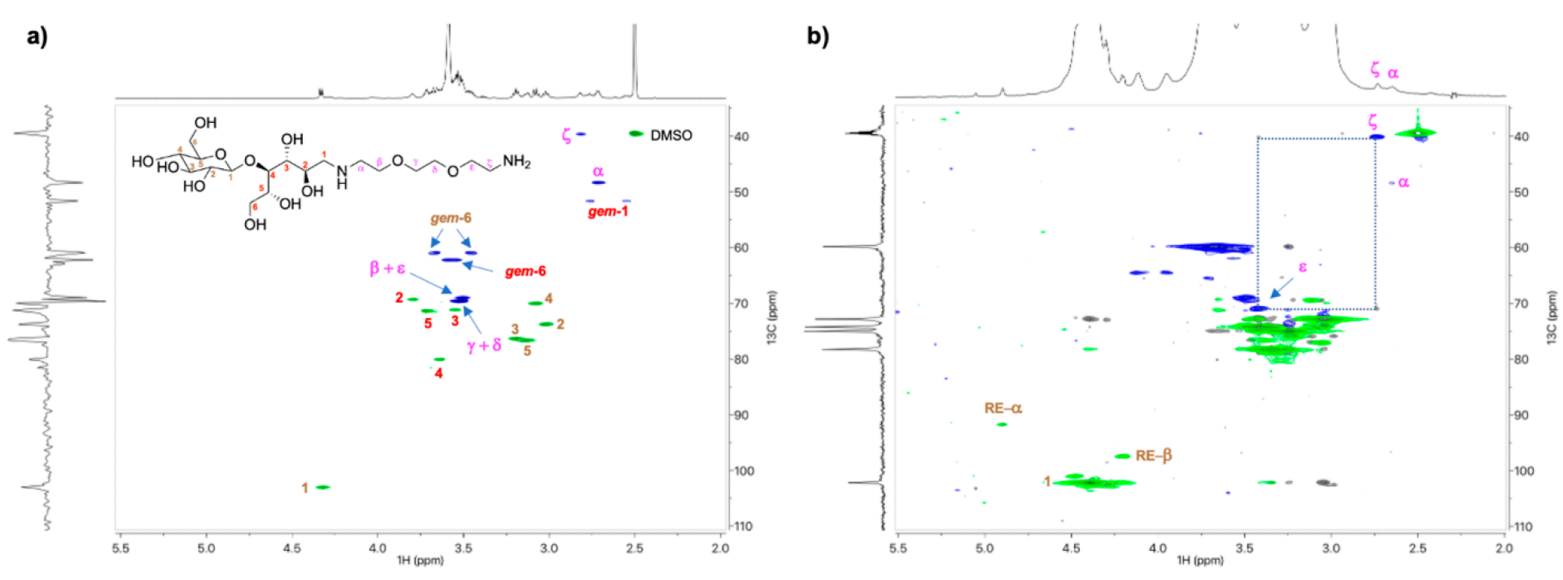
(a) Multiplicity-edited HSQC spectrum of cellobiose-diamine M2 reductive amination conjugate (in DMSO-*d*_*6*_ at 65 °C), and (b) HSQC-TOCSY spectrum (15 ms TOCSY mixing time) with multiplicity-edited HSQC overlay and diffusion-edited ^1^H trace of CNC-RE-*g*-NH_2_ reductive amination conjugate (in [P_4444_][OAc]/DMSO-*d*_6_ at 65 °C); the ζ to ε TOCSY correlation is shown with the dotted line box.

Based on our observation that the initiator at least partially decomposes during reaction pathway 1, the two-step approach (Scheme 1) has the potential benefit that the ATRP-initiator is attached as an activated ester (NHS-BiBB) to amino-modified REGs under very mild reaction conditions, possibly avoiding its passivation. Thus, in the first reaction step, 2,2′-(ethylenedioxy)bis(ethylamine) was attached to the CNC REGs to form CNC-RE-*g*-NH_2_ using similar reductive amination conditions as employed for CNC-RE-*g*-BiBB-1. The BCA assay showed a higher conversion (ca. 88%) than to the first reaction route (<60%; Table 1), and the concentration of aldehyde groups was reduced from 16 μmol CHO/g CNC to 2 μmol CHO/g CNC. The BCA assay showed a higher conversion (ca. 88%), compared to the first reaction route (<60%; Table 1), and the concentration of aldehyde groups was reduced from 16 μmol CHO/g of CNCs to 2 μmol CHO/g of CNCs. Furthermore, to rule out the adsorption of the diamine on the surface of the CNCs, a blank test was performed on the CNCs that were oxidized at their REGs (CNC-RE-COOH), as these CNCs would not be able to undergo a reductive amination with a diamine; they do not possess aldehyde groups at the CNC extremity. The ninhydrin assay confirmed that there is little to no adsorption of the diamine on the surface of the CNC-RE-COOH (4 μmol NH_2_ /g CNC), after multiple purification steps. Thus, we can conclude that the amines quantified by the ninhydrin assay, for CNC-RE-*g*-NH_2_, are not adsorbed but rather attached to the CNCs. NMR analysis confirmed the attachment of the diamine to the REGs of both the cellobiose model (M2, Figure 2a) and CNCs (Figure 2b). Full details on the compound purification and resonance assignment for M2 can be found in the Supporting Information, Figure S7.

When performing the reductive amination on the CNCs, to yield CNC-RE-*g*-NH_2_, the presence of the appended amines was clearly apparent in the diffusion-edited ^1^H spectrum (Figure 2b, ^1^H trace). In addition, the HSQC-TOCSY in [P_4444_][OAc]/DMSO-*d*_6_ also showed the presence of the ζ, α, and ε resonances, with a clear TOCSY correlation between the ζ and ε CH_2_ moieties on the amine tail. Thus, this clearly demonstrates the efficiency of the reductive amination conditions.

In the second reaction step of the two-step pathway, the amino-functionalized CNC-RE-*g*-NH_2_ was reacted with NHS-BiBB affording CNC-RE-*g*-BiBB-2 (Scheme 1). The concentration of primary amine groups remaining after this reaction was quantified by the ninhydrin assay, which demonstrated a reduction in the concentration of primary amines from 21 μmol NH_2_/g CNC to 5 μmol NH_2_/g CNC. Our preliminary conclusions were that the reaction took place but was not complete.

**Figure 3:**
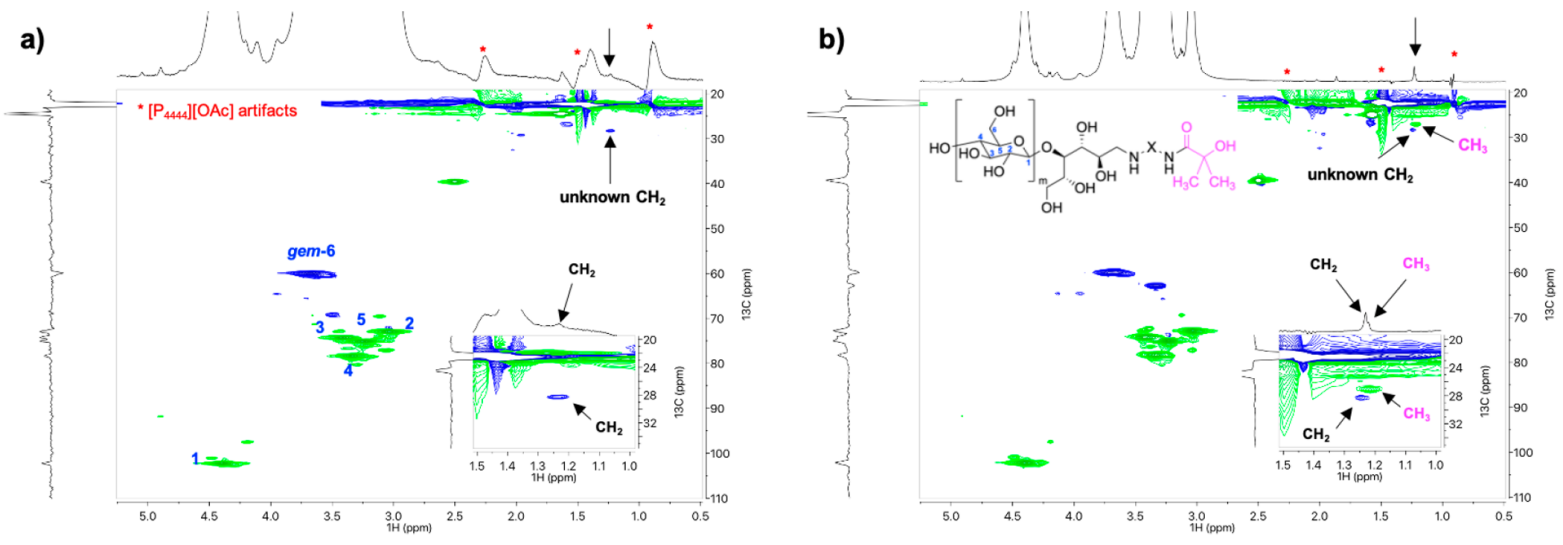
Multiplicity-edited HSQC spectra (in [P_4444_][OAc]/DMSO-*d*_6_ at 65 °C, with the diffusion-edited ^1^H trace) for (a) CNC-RE-*g*-BiBB-2 and (b) CNC-RE-*g*-BiBB-1. Signals associated with CH and CH_3_ groups are shown in green and CH_2_ signals are shown in blue. The red stars indicate the peaks assigned to the [P_4444_][OAc] artifacts.

In Figure 3a, the 2D NMR spectra and assignments for the BiBB moiety in CNC-RE-*g*-BiBB-2 are shown. The spectra of M2 and M3(Figure 2a and Supporting Information, Figures S7 and S9) were used for the assignment of the signals associated with the ATRP-initiator. In the diffusion-edited ^1^H trace, a clear signal was observed at 1.22 ppm, matching the expected chemical shift region for the BiBB-OH terminal CH_3_ groups. However, the multiplicity-edited HSQC identified the multiplicity of this signal to be a CH_2_, arising from an unknown species (does not correlate with any expected species, based on the sample history and chemistry applied). The spectrum was further compared to the one of CNC-RE-*g*-BiBB-1 (Figure 3b), which was produced via route 1 (one-step pathway). In this case, the unknown CH_2_ signal was also present, except that it was adjacent to an additional signal of the correct multiplicity (CH_3_). Moreover, HMBC correlations for this CH_3_ (Figure 1a) showed connectivity (with the correct ^13^C chemical shifts) to a quaternary carbon (71.6 ppm) and a carbonyl (amide, 176.5 ppm), as were observed for the BiBB-OH form of amino-functionalized ATRP-initiator-1 (Figure 1g) and the BiBB-OH form of M1 (Figure 1b).

The comparison with the spectrum of CNC-RE-*g*-BiBB-1, and the absence of the terminal CH_3_ signal in the diffusion-edited ^1^H traces suggested that the functionalization of CNC-RE-*g*-NH_2_ with the NHS-BiBB did not occur to a measurable extent. Furthermore, methacrylamide signals (from BiBB-Br in the NMR media) were also missing. In the case of the cellobiose model M3, only a partial conversion was observed after the 4 h reaction as the mass spectra of M3 (Supporting Information, Figure S10) exhibited both signals of the product and the cellobiosylamine M2.

When comparing the two pathways toward the selective introduction of BiBB to CNC REGs, one can conclude that the one-step pathway is more straightforward, since no intermediate purification is needed. However, passivation of the initiator was observed, likely as a side-reaction in water at elevated temperature and in acidic pH.^49^ The two-step pathway is more time-consuming due to the need for intermediate purification. Nevertheless, the conversion of the REGs with the diamine under the chosen reductive amination conditions was efficient. Furthermore, it had the potential advantage to attach BiBB at RT, avoiding its passivation. Yet the second reaction step had a nonobservable conversion by NMR analysis. This may be due to the hydrolysis of NHS-BiBB in water, although, this is not consistent with the ninhydrin analysis, suggesting a consumption of amine. Additionally, when dissolving CNC-RE-*g*-BiBB-2 in the NMR solvent, the sample did not completely clarify, indicating that a portion of the material did not dissolve. Extended heating at 65 °C did not resolve this problem, which was indicative of some kind of aggregation, as a result of covalent (chemical) cross-linking. This could have the effect of rendering the introduced resonances invisible to solution-state NMR. The mechanism of this potential cross-linking is a mystery, although reactions of hindered amines with BiBB-Br moieties (nucleophilic substitution of bromine with amine), yielding secondary amines are known.^51^ Further work is required to understand this phenomenon, but it is clear this route contains challenges.

### Grafting from the CNC REGs, Characterization, and Properties

**Scheme 2:**
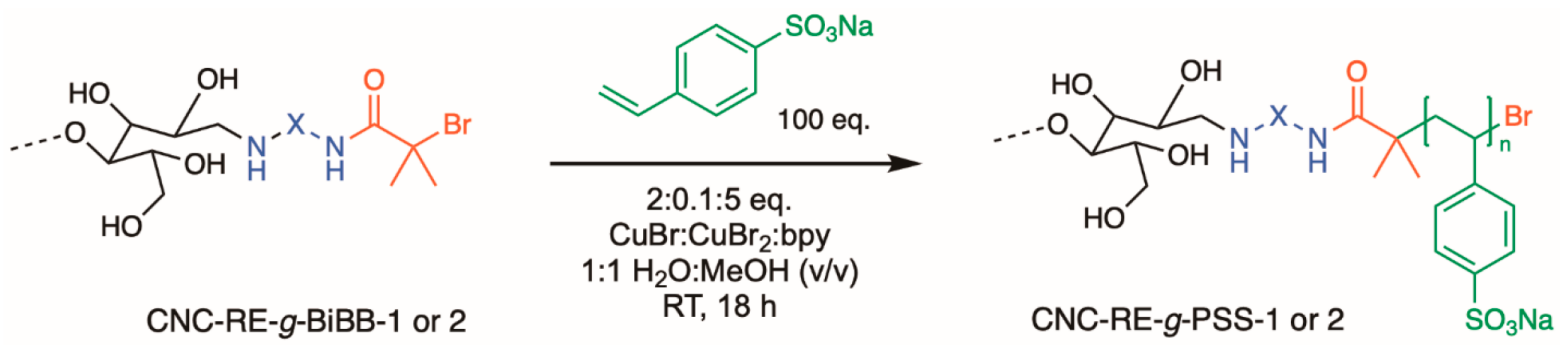
Reaction Scheme in Which Grafting of PSS from CNC-RE-*g*-BiBB-1 and -2 Is Shown

In spite of the low REG-modification with the ATRP-initiator, an anionic polyelectrolyte, poly(sodium 4-styrenesulfonate) (PSS), was grafted from CNCs modified with initiators following both routes 1 and 2, resulting in CNC-RE-*g*-PSS-1 and CNC-RE-*g*-PSS-2 (Scheme 2), respectively. Additionally, a low-molecular-weight PSS homopolymer, initiated with ethyl α-bromoisobutyrate, was synthesized, which exhibited distinctive FT-IR absorption bands at 1180, 1128, 1040, and 1009 cm^–1^. In Figure S18, the FT-IR spectra of the different CNCs are shown, the PSS bands overlap in most regions with the CNC absorption bands, which made the characterization of the PSS grafts using IR difficult. Therefore, the peak intensity at 1180 cm^–1^, which corresponds to the antisymmetric vibrational adsorption peaks of the sulfonate group,^45^ was compared to the intensity of the signal at 1424 cm^–1^, which corresponds to the CH_2_ scissor motion in cellulose.^52^ The ratio between the intensity of these bands for unmodified CNCs was 0.9, and increased to 1.1 for both CNC-RE-*g-*PSS samples, suggesting that PSS was introduced to the CNCs.

More direct evidence of the presence of PSS was given by elemental analysis (EA; Table 2). The modified CNCs demonstrated an increase in sulfur content from 0.8% for the unmodified CNCs to 2.6% (CNC-RE-*g*-PSS-1) and 5.5% (CNC-RE-*g*-PSS-2), indicating a successful growth of PSS from the REGs. The higher sulfur content determined for CNC-RE-*g*-PSS-2 is somewhat surprising as the BiBB signals were absent in the NMR spectrum. This presents two options, for which we can only speculate at this point: (1) The ATRP-initiator might not have been visible in solution-state NMR due to irreversible (covalent) aggregation of the sample, as discussed above; or (2) The incorporated initiator was very low, in reality, but the graft molecular weights are very high, resulting in higher sulfur contents. In addition to this ambiguity, more work would need to be done to be able to accurately quantify the degree of passivation of the ATRP initiators, for any sample, to allow for further accurate calculation of graft density and length from NMR. A small increase in the nitrogen content compared to the unmodified CNCs was observed, likely corresponding to the introduction of the diamine. Yet, the increase falls within the detection limit of the instrument and, thus, cannot give conclusive results. Unfortunately, bromine quantification was also not possible as a result of the detection limit of the instrument.

**Table 2:** Elemental Compositions for Unmodified CNCs, PSS Grafted CNCs via the First Reaction Route (CNC-RE-*g*-PSS-1) and PSS Grafted CNCs via the Second Reaction Route (CNC-RE-*g*-PSS-2)

	C (%)	H (%)	N (%)	S (%)
unmodified CNCs	40.9 ± 0.1	5.9 ± 0.0	0.2 ± 0.1	0.8 ± 0.1
CNC-RE-*g*-PSS-1	41.1 ± 0.0	5.6 ± 0.1	0.4 ± 0.1	2.6 ± 0.1
CNC-RE-*g*-PSS-2	41.0 ± 0.1	5.8 ± 0.1	0.3 ± 0.1	5.5 ± 0.1

The apparent particle size of the CNCs before and after PSS grafting, and their colloidal stability in aqueous dispersion was evaluated using DLS (Table S2). These measurements were performed on dilute CNC suspensions in their acid form and contained 0.5 mM NaCl. They were evaluated under the assumption of spherical particles; as such, they represent the apparent size of CNCs in suspension rather than a specific dimension (e.g., particle length). The apparent size increased from 62 ± 0.2 nm for the unmodified CNCs to 68 ± 0.2 and 111 ± 0.7 nm for CNC-RE-*g*-BiBB-1 and CNC-RE-*g*-BiBB-2, respectively, and 123 ± 0.2 and 126 ± 0.3 nm for the PSS-grafted CNCs after modification via routes 1 and 2, respectively. The higher apparent size obtained for CNC-RE-*g*-BiBB-2 is likely the result of particle aggregation of the modified CNCs, as suggested above. However, the increase after grafting of PSS is some indication that the suspension properties changed. In addition, AFM was performed after modification (Supporting Information, Figure S19), and the images show that the rod-like morphology of the CNCs was preserved after modification.

ζ-Potential measurements of the modified CNCs in their acid form (containing 5 mM of NaCl) showed a decrease from −34 ± 1 mV for unmodified CNCs to −37 ± 2 mV (for CNC-RE-*g*-PSS-1) and −41 ± 1 mV (for CNC-RE-*g*-PSS-2). In addition, a blank reaction was ran with CNCs at the conditions used during the reductive amination; no change in ζ-potential was measured after this reaction. We emphasize that these values should only be taken as relative measures, because the ζ-potential calculation from the electrophoretic movement has inherent assumptions that are not met for CNCs (e.g., shape, size, and charge density).^53,54^ Nevertheless, the end-grafted CNCs appear well-dispersed by DLS and are colloidally stable in water at the measured concentration (0.25 wt %), after grafting of PSS from the REGs of the CNCs.

**Figure 4:**
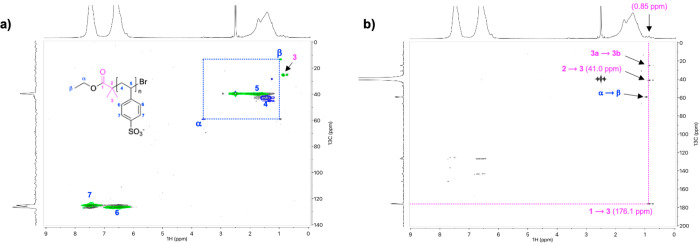
HSQC and HMBC NMR spectra of the PSS homopolymer initiated with EBiB (in DMSO-*d*_6_ at 65 °C): (a) HSQC-TOCSY (15 ms TOCSY mixing time for short-range correlations) and (b) ^13^C HMBC.

The successful grafting of PSS from the CNC REGs was further examined by NMR analysis using the PSS homopolymer as a model to assign the signals associated with the initiator–graft linkage point in the NMR spectra. In Figure 4 the data and HSQC assignments for the homopolymer are shown, with ^13^C HMBC correlations to the BiBB moiety 3-CH_3_ positions (Figure 4b). The HMBC correlations for the expected BiBB linker 1, 2, and 3 positions are clearly distinct, as is the ^1^H peak position for the 3-CH_3_ groups (0.85 ppm). These aforementioned resonances and associated 2D correlations were further used to distinguish the signals associated with the BiBB initiator, its decomposition products, and its presence as a linker for grafting.

**Figure 5:**
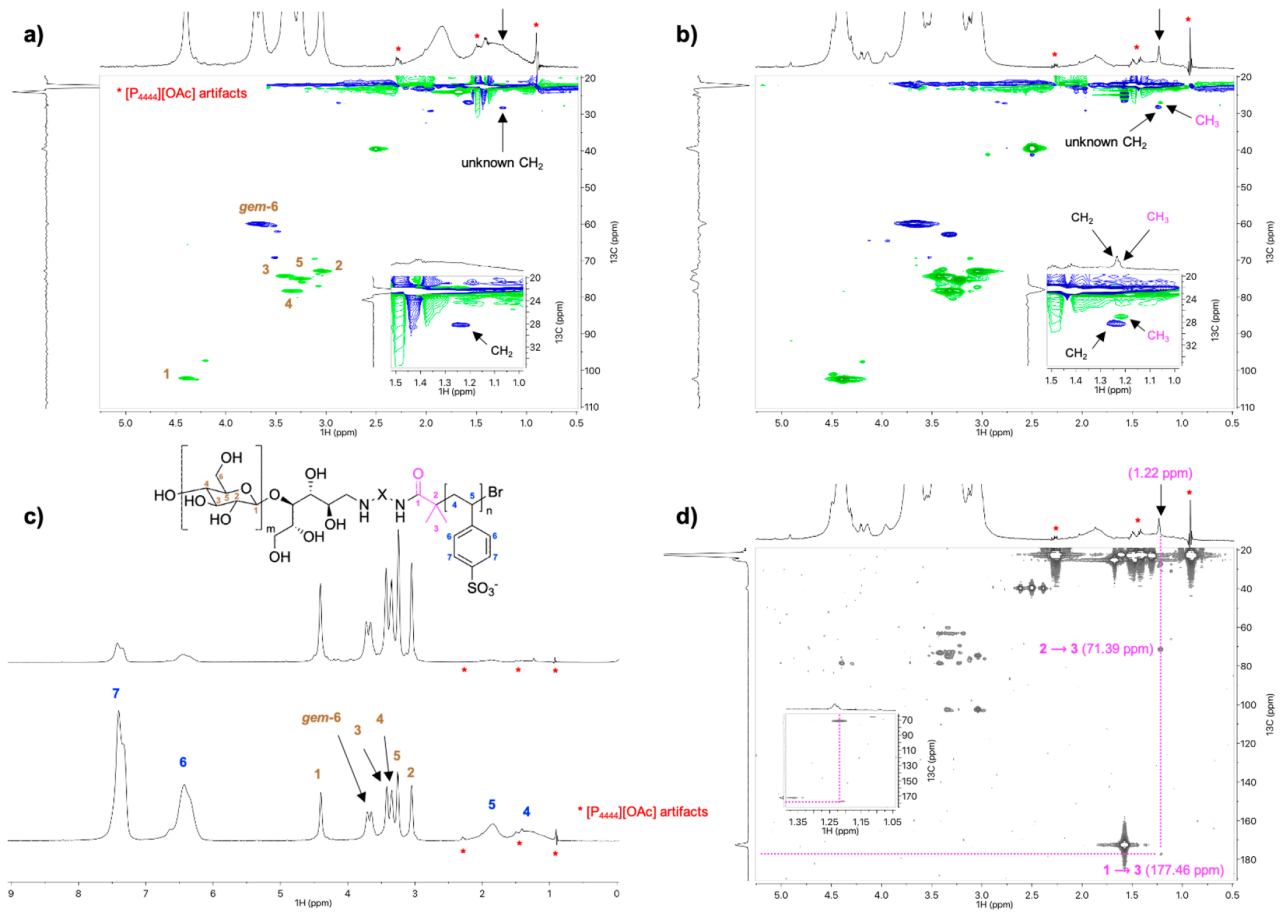
(a) Multiplicity-edited HSQC spectrum of CNC-RE-*g*-PSS-2 (in [P_4444_][OAc]/DMSO-*d*_6_ at 65 °C, with the diffusion-edited ^1^H trace); (b) Multiplicity-edited HSQC spectrum of CNC-RE-*g*-BiBB-1 (in [P_4444_][OAc]/DMSO-*d*_6_ at 65 °C, with the diffusion-edited ^1^H trace); (c) Diffusion-edited ^1^H spectra of CNC-RE-*g*-PSS-1 (top) and CNC-RE-*g*-PSS-2 (bottom) (in [P_4444_][OAc]/DMSO-*d*_6_ at 65 °C); (d) HMBC of CNC-RE-*g*-PSS-1 (in [P_4444_][OAc]/DMSO-*d*_6_ at 65 °C).

HSQC NMR spectra of the grafted CNC-RE-*g*-PSS products are demonstrated in Figure 5. The diffusion-edited ^1^H spectra (Figure 5c) clearly revealed the presence of PSS and cellulose, for CNC-RE-*g*-PSS-2 (Figure 5c, top) and CNC-RE-*g*-PSS-1 (Figure 5c, bottom). Thus, the passivation of the initiator in both cases was incomplete and ATRP polymerization could still take place. Baseline correction and peak fitting of the cellulose 1–6 signals versus the PSS aromatic signals in the ^1^H spectra for the CNC-RE-*g*-PSS samples (Supporting Information, S4e) yielded a PSS content of 16 ± 5 wt % for CNC-RE-*g*-PSS-1 and 67 ± 5 wt % PSS for CNC-RE-*g*-PSS-2. The higher concentration of PSS incorporation in CNC-RE-*g*-PSS-2 is consistent with the elemental analysis. While the values obtained from these spectra and processing strategy should be viewed as semiquantitative, they are more accurate than other available methods. Using the values obtained via EA, a PSS weight fraction of 9 and 25 wt % was calculated for CNC-RE-*g*-PSS-1 and -2, respectively. The differences between EA and NMR may be explained by the lower accuracy of elemental analysis, possibly due to the poor combustion of the PSS salt portion (which has a very large content of Na^+^ cations), as the analysis was performed on CNC-RE-*g*-PSS in sodium form.^55^ Therefore, the sulfur content is likely underestimated, and these results should merely be used for qualitative arguments, without EA optimization with suitable standards.

Furthermore, a tentative molecular weight (MW) of the PSS grafts was estimated based on the disappearance of REGs, followed by attachment of ATRP initiators. Based on the results of the colorimetric methods used to estimate the attachment of the ATRP initiators (Table 1) and the PSS concentrations deduced from NMR analysis, the DP of the PSS grafts was estimated. Accordingly, CNC-RE-*g*-PSS-1 and CNC-RE-*g*-PSS-2 had an estimated DPn of 87 (MW = 18000 g/mol) and 303 (MW = 62500 g/mol), respectively. As the passivation of the initiator cannot be ruled out (Figure 1), the number of active initiators is likely lower, which would suggest an underestimated DP, yet they represent a reliable estimate of the minimum PSS MWs obtained.

In the BiBB-OH CH_3_ chemical shift region, the multiplicity-edited HSQC for CNC-RE-*g*-BiBB-2 (Figure 5a) only showed the signal of the unassigned CH_2_ group, as observed for the previous steps (reductive amination and BiBB introduction). The multiplicity-edited HSQC for CNC-RE-*g*-BiBB-1 (Figure 5b) showed the presence of the CH_3_ for BiBB-OH (further discussed above in Side Reactions of the ATRP-Moieties). This was corroborated by the corresponding HMBC correlations to the 3 position, expected for BiBB-OH, with the correct ^13^C chemical shift positions for the correlations. Unfortunately, none of these spectra showed the presence of a linking BiBB moiety to the polymer graft, as assigned in Figure 4. However, this does not mean that those linking points were not present. It is not unexpected that linking points of this kind will demonstrate fast *T*_2_ relaxation, such that they will no longer be visible in HMBC especially due to restricted motion. The expected ^1^H signal positions were also somewhat overlapping with the [P_4444_][OAc] terminal CH_3_ signal and artifacts in the ^1^H and diffusion-edited ^1^H spectra, respectively. Thus, with this initiator it may not be possible to directly identify these linkage points using solution-state NMR.

**Figure 6:**
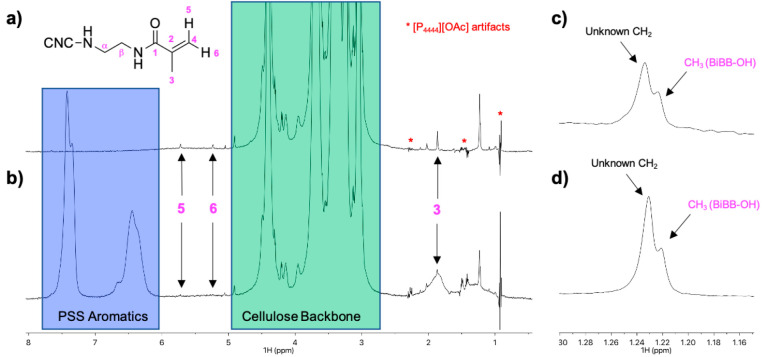
Normalized diffusion-edited ^1^H spectra (in [P_4444_][OAc]/DMSO-*d*_6_ at 65 °C) for (a) CNC-RE-*g*-BiBB-1, (b) CNC-RE-*g*-PSS-1, (c) 1.15–1.30 ppm expansion for CNC-RE-*g*-BiBB-1, and (d) 1.15–1.30 ppm expansion for CNC-RE-*g*-PSS-1.

Considering this, the final option for investigating the conversion of initiators to PSS grafts using NMR spectroscopy lies with the rapid degradation of residual BiBB-Br moieties to methacrylamide in the [P_4444_][OAc]/DMSO-*d*_6_ electrolyte. A comparison of the normalized (cellulose backbone signals) diffusion-edited ^1^H spectra (Figure 6) for CNC-RE-*g*-BiBB-1 and CNC-RE-*g*-PSS-1 (before and after grafting) showed that the intensity of the signal assigned to the BiBB-OH CH_3_ groups experienced only a small change upon reaction (Figure 6c,d). This is consistent with initiator passivation to BiBB-OH during the reductive amination reaction. However, signals for methacrylamide were clearly present in the diffusion-edited ^1^H spectrum for CNC-RE-*g*-BiBB-1 (Figure 6a), indicating the presence of BiBB-Br, as well as BiBB-OH. Furthermore, the intensity of the methacrylamide signals was significantly reduced in the spectrum for CNC-RE-*g*-PSS-1, indicating that a large portion of BiBB-Br (which was not pacified) was consumed during the polymerization. This is indirect but strong evidence of REG grafting. Similar spectra for CNC-RE-*g*-BiBB-2 and CNC-RE-*g*-PSS-2 (Supporting Information, Figure S17) did not show the presence of methacrylamide signals.

Based on the hypothesis that the PSS end-tethered CNCs will retain their colloidal stability in water and their electrostatic repulsion, which is important for their liquid crystalline self-assembly, the liquid crystalline properties of the CNCs were investigated. Suspensions of never-dried PSS grafted CNCs-RE-*g*-PSS-1 and CNCs-RE-*g*-PSS-2 (in acid form after SAC column) in water exhibited shear birefringence, as expected for anisotropic colloids that are colloidally stable. Furthermore, after freeze-drying the CNC-RE-*g*-PSS in acidic form, they could be easily redispersed in water and retained their shear birefringence (Supporting Information, Figure S20), which is not the case for unmodified sulfated CNCs.^56^ The CNCs were rendered slightly more amphiphilic after modification as shown by their surface tension measured via the pendant drop method. The surface tension of the CNCs decreased from 75 ± 0.5 mN/m for unmodified CNCs to 73 ± 0.6 mN/m for CNCs-RE-*g*-PSS-1 and 70 ± 0.4 mN/m for CNCs-RE-*g*-PSS-2, again demonstrating a change after modification, but not enough to be surface-active.

Sufficiently concentrated, aqueous suspensions of CNCs are known to separate into an isotropic upper phase and a birefringent lower phase.^57^ Indeed, the unmodified CNC suspensions containing 1 mM NaCl phase-separated above a critical concentration of 7 wt %. However, the modified CNCs could not be concentrated up to this level, but instead formed gels before reaching that concentration (above 4 wt % for CNC-RE-*g*-PSS-1 and 6 wt % for CNC-RE-*g*-PSS-2). As expected, suspensions with lower concentrations of modified CNCs (4 wt % for CNC-RE-*g*-PSS-1 and 5 wt % for CNC-RE-*g*-PSS-2, containing 1 mM NaCl) did not phase-separate (Supporting Information, Figure S21). In the case of CNC-RE-*g*-PSS-2, which contains a higher fraction of PSS, the suspension turned white instead of presenting the typical blueish hue observed for unmodified CNCs. Furthermore, a water layer was formed at the top of the capillary. The increase in concentration likely caused the CNC-RE-*g*-PSS-2 to aggregate and become colloidally unstable. If CNC-RE-*g*-PSS-2 samples indeed have a higher molecular weight of the end-tethered grafts compared to CNC-RE-*g*-PSS-1, grafted PSS chain entanglements between adjacent CNCs could have inhibited liquid crystal phase formation, which highlights the importance of the graft length on the liquid crystalline behavior of CNCs.

**Figure 7:**
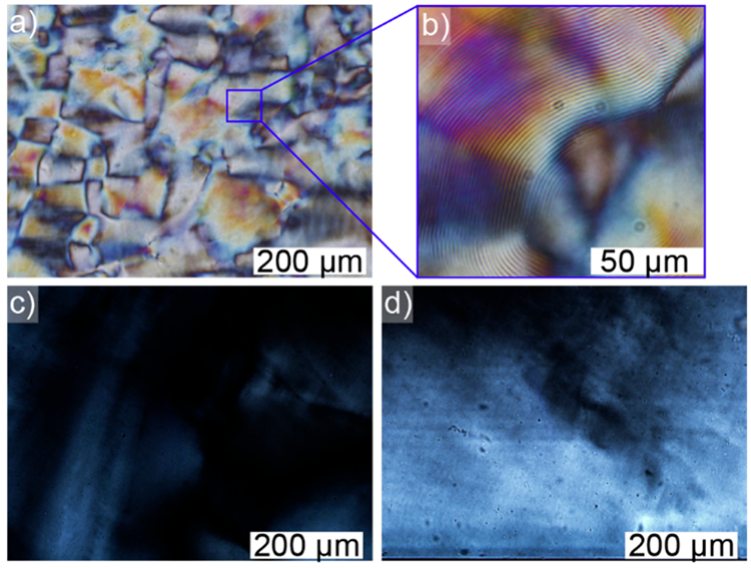
Polarized optical microscopy image of flat capillary tubes filled with concentrated CNC suspensions: (a) An aqueous suspension containing 9 wt % of unmodified CNCs (and 1 mM of NaCl); (b) Image of (a), demonstrating chiral nematic fingerprint structures; c) An aqueous suspension containing 4 wt % of CNC-RE-*g*-PSS-1 (and 1 mM NaCl) demonstrating some minor birefringence at the top of the capillary; (d) A capillary of a 5 wt % CNC-RE-*g*-PSS-2 (and 1 mM NaCl), showing a nematic texture.

The unmodified CNC suspension (9 wt %, 1 mM NaCl) exhibited the expected chiral nematic fingerprint structures above its critical concentration.^37,58^ CNC-RE-*g*-PSS-1 demonstrated no ordering at the concentration studied (4 wt %), except at the top of the capillary where faint birefringence could be observed. However, a nematic structure was observed in the case of CNC-RE-*g*-PSS-2 (5 wt %, 1 mM NaCl; Figure 7). We hypothesize that the end-tethered macromolecules interfered with the self-assembly of the CNCs by changing the apparent nanocrystal aspect ratio and the resulting order parameter. Previously we observed a similar disruption of the chiral nematic liquid crystalline structure by grafting poly(*N*-isopropylacrylamide), preferentially from the REGs of the CNCs.^27^ The CNC-RE-*g*-PNIPAM exhibited birefringence, but no chiral nematic ordering at both room and elevated temperatures. A second possibility is that the CNC-RE-*g*-PSS reached a kinetically arrested gel-like phase, prohibiting the modified CNCs to reach the critical concentration needed to form a chiral nematic liquid crystalline phase.^57^ These effects, as a result of asymmetric grafting, are intriguing and more research is needed to study the impact of the graft length on the properties of these Janus-type CNC hybrids, in terms of their self-assembly behavior. Their assembly might give rise to totally new 3D nanostructures whose architectures are controllable based on graft polymer selection, as well as graft length and density.^12^

## Conclusions

Two pathways to selectively end-modify CNCs with ATRP-initiators were studied: (1) a direct pathway using reductive amination to attach an amino-terminated ATRP-initiator, and (2) a two-step pathway using reductive amination followed by NHS-mediated coupling of the polymerization initiator. Significant limitations were observed for both pathways. CNCs modified via route 1 demonstrated an overall higher conversion compared to route 2 according to NMR analysis. However, the elevated temperatures required for reductive amination resulted in a partial passivation of REG-grafted ATRP-initiator, to the detriment of the later polymerization. Furthermore, PSS was grafted from the CNC REGs, which was confirmed by consumption of the portion of initiator that was not passified.

While the two-step pathway looked good on paper, we could not confirm grafting of the polymer at the REG, despite a covalently attached polymer being present. After initiator introduction, the sample showed some partial insolubility, indicating irreversible aggregation, that significantly affected the intended properties of the material. The obtained Janus-type nanocellulose hybrids were colloidally stable at low concentrations, they maintained shear birefringence after freeze-drying/redispersion and were more easily redispersed in water than unmodified CNCs (in acid form). Their asymmetric character could thereby lead to totally new 3D-nanostructures with anisotropic properties. The current study demonstrates the importance of combining different analytical techniques to confirm, quantify, and assess the end-group modification of CNCs. Furthermore, polymer grafting from the REGs was confirmed to a high degree of accuracy, in the absence of direct microscopy-based confirmation. Future work could involve grafting of a photocleavable ATRP-initiator, as previously demonstrated by Morandi et al.,^59^ in order to fully characterize the grafted polymers, which would give further insight in the effect of polymer length on the self-assembly behavior of CNCs.

## Supporting Information Available

The characterization of the pristine CNCs, additional information and spectra on the cellobiose model compounds, the ATRP-initiator stability in D_2_O, the NMR methods and remaining NMR data, and the characterization of the polymer-grafted CNCs (PDF)

## Abbreviations

AGU: anhydrous glucose unit
BiBB: α-bromoisobutyryl bromide
bpy: 2,2′-bipyridine
CNC: cellulose nanocrystal
DI: deionized
DLS: dynamic light scattering
DP: degree of polymerization
EA: elemental analysis
EBEA: 2,2′-(ethylenedioxy)-bis(ethylamine)
EBiB: ethyl α-bromoisobutyrate
HRMS: high-resolution mass spectrometry
HMBC: Heteronuclear Multiple Bond Correlation
HSQC: Heteronuclear Single Quantum Correlation
LC: liquid chromatography
MW: molecular weight
MWCO: molecular weight cutoff
NaPSS: polystyrenesulfonate sodium salt
NHS: *N*-hydroxysuccinimide
2-PCB: 2-picolineborane
PNIPAM: poly(*N*-isopropylacrylamide)
POM: polarized optical microscopy
RE: reducing end
REG: reducing end-group
RT: room temperature
4-SS: 4-vinylbenzyl sulfonate
SI-ATRP: surface-initiated atom-transfer radical polymerization
TLC: thin-layer chromatography
TOCSY: Total Correlation Spectroscopy

## References

[ref1] Trache D.; Hussin M. H.; Haafiz M. K. M.; Thakur V. K. Recent Progress in Cellulose Nanocrystals: Sources and Production. Nanoscale 2017, 9 (5), 1763–1786. 10.1039/C6NR09494E. 28116390

[ref2] Abitbol T.; Kloser E.; Gray D. G. Estimation of the Surface Sulfur Content of Cellulose Nanocrystals Prepared by Sulfuric Acid Hydrolysis. Cellulose 2013, 20 (2), 785–794. 10.1007/s10570-013-9871-0.

[ref3] Dong S.; Bortner M. J.; Roman M. Analysis of the Sulfuric Acid Hydrolysis of Wood Pulp for Cellulose Nanocrystal Production: A Central Composite Design Study. Ind. Crops Prod. 2016, 93, 76–87. 10.1016/j.indcrop.2016.01.048.

[ref4] Shatkin J. A.H.; Wegner T.; Bilek E. M.; Cowie J. Market Projections of Cellulose Nanomaterial-Enabled Products? Part 1: Applications. Tappi J. 2014, 13 (5), 9–16. 10.32964/TJ13.5.9.

[ref5] Kontturi E.; Laaksonen P.; Linder M. B.; Nonappa; Groschel A. H.; Rojas O. J.; Ikkala O. Advanced Materials through Assembly of Nanocelluloses. Adv. Mater. 2018, 30 (24), 1703779 10.1002/adma.201703779. 29504161

[ref6] Vanderfleet O. M.; Cranston E. D. Production Routes to Tailor the Performance of Cellulose Nanocrystals. Nat. Rev. Mater. 2021, 6, 124 10.1038/s41578-020-00239-y.

[ref7] Heise K.; Kontturi E.; Allahverdiyeva Y.; Tammelin T.; Linder M. B.; Nonappa; Ikkala O. Nanocellulose: Recent Fundamental Advances and Emerging Biological and Biomimicking Applications. Adv. Mater. 2021, 33 (3), 2004349 10.1002/adma.202004349. PMC11468234 33289188

[ref8] Habibi Y. Key Advances in the Chemical Modification of Nanocelluloses. Chem. Soc. Rev. 2014, 43 (5), 1519–1542. 10.1039/C3CS60204D. 24316693

[ref9] Eyley S.; Thielemans W. Surface Modification of Cellulose Nanocrystals. Nanoscale 2014, 6 (14), 7764–7779. 10.1039/C4NR01756K. 24937092

[ref10] Wohlhauser S.; Delepierre G.; Labet M.; Morandi G.; Thielemans W.; Weder C.; Zoppe J. O. Grafting Polymers *from* Cellulose Nanocrystals: Synthesis, Properties, and Applications. Macromolecules 2018, 51 (16), 6157–6189. 10.1021/acs.macromol.8b00733.

[ref11] Kedzior S. A.; Zoppe J. O.; Berry R. M.; Cranston E. D. Recent Advances and an Industrial Perspective of Cellulose Nanocrystal Functionalization through Polymer Grafting. Curr. Opin. Solid State Mater. Sci. 2019, 23 (2), 74–91. 10.1016/j.cossms.2018.11.005.

[ref12] Heise K.; Delepierre G.; King A. W. T.; Kostiainen M. A.; Zoppe J.; Weder C.; Kontturi E. Chemical Modification of Reducing End-Groups in Cellulose Nanocrystals. Angew. Chem., Int. Ed. 2021, 60 (1), 66–87. 10.1002/anie.202002433. PMC7821002 32329947

[ref13] Tao H.; Lavoine N.; Jiang F.; Tang J.; Lin N. Reducing End Modification on Cellulose Nanocrystals: Strategy, Characterization, Applications and Challenges. Nanoscale Horiz. 2020, 5 (4), 607–627. 10.1039/D0NH00016G. 32073114

[ref14] Koyama M.; Helbert W.; Imai T.; Sugiyama J.; Henrissat B. Parallel-up Structure Evidences the Molecular Directionality during Biosynthesis of Bacterial Cellulose. Proc. Natl. Acad. Sci. U. S. A. 1997, 94 (17), 9091–9095. 10.1073/pnas.94.17.9091. 9256440 PMC23045

[ref15] Lin F.; Putaux J.-L.; Jean B. Optimized Reducing-End Labeling of Cellulose Nanocrystals: Implication for the Structure of Microfibril Bundles in Plant Cell Walls. Carbohydr. Polym. 2021, 257, 117618 10.1016/j.carbpol.2021.117618. 33541646

[ref16] Walther A.; Müller A. H. E. Janus Particles: Synthesis, Self-Assembly, Physical Properties, and Applications. Chem. Rev. 2013, 113 (7), 5194–5261. 10.1021/cr300089t. 23557169

[ref17] Su H.; Hurd Price C.-A.; Jing L.; Tian Q.; Liu J.; Qian K. Janus Particles: Design, Preparation, and Biomedical Applications. Mater. Today Bio 2019, 4, 100033 10.1016/j.mtbio.2019.100033. PMC7061647 32159157

[ref18] Safaie N.; Ferrier R. C. Janus Nanoparticle Synthesis: Overview, Recent Developments, and Applications. J. Appl. Phys. 2020, 127 (17), 170902 10.1063/5.0003329.

[ref19] Marschelke C.; Fery A.; Synytska A. Janus Particles: From Concepts to Environmentally Friendly Materials and Sustainable Applications. Colloid Polym. Sci. 2020, 298 (7), 841–865. 10.1007/s00396-020-04601-y.

[ref20] King A. W. T.; Mäkelä V.; Kedzior S. A.; Laaksonen T.; Partl G. J.; Heikkinen S.; Koskela H.; Heikkinen H. A.; Holding A. J.; Cranston E. D.; Kilpeläinen I. Liquid-State NMR Analysis of Nanocelluloses. Biomacromolecules 2018, 19 (7), 2708–2720. 10.1021/acs.biomac.8b00295. 29614220

[ref21] Koso T.; Rico del Cerro D.; Heikkinen S.; Nypelö T.; Buffiere J.; Perea-Buceta J. E.; Potthast A.; Rosenau T.; Heikkinen H.; Maaheimo H.; Isogai A.; Kilpeläinen I.; King A. W. T. 2D Assignment and Quantitative Analysis of Cellulose and Oxidized Celluloses Using Solution-State NMR Spectroscopy. Cellulose 2020, 27 (14), 7929–7953. 10.1007/s10570-020-03317-0.

[ref22] Heise K.; Koso T.; Pitkänen L.; Potthast A.; King A. W. T.; Kostiainen M. A.; Kontturi E. Knoevenagel Condensation for Modifying the Reducing End Groups of Cellulose Nanocrystals. ACS Macro Lett. 2019, 8 (12), 1642–1647. 10.1021/acsmacrolett.9b00838. 35619387

[ref23] Lokanathan A. R.; Nykänen A.; Seitsonen J.; Johansson L.-S.; Campbell J.; Rojas O. J.; Ikkala O.; Laine J. Cilia-Mimetic Hairy Surfaces Based on End-Immobilized Nanocellulose Colloidal Rods. Biomacromolecules 2013, 14 (8), 2807–2813. 10.1021/bm400633r. 23799635

[ref24] Yang H.; Alam Md. N.; van de Ven T. G. M. Highly Charged Nanocrystalline Cellulose and Dicarboxylated Cellulose from Periodate and Chlorite Oxidized Cellulose Fibers. Cellulose 2013, 20 (4), 1865–1875. 10.1007/s10570-013-9966-7.

[ref25] Arcot L. R.; Lundahl M.; Rojas O. J.; Laine J. Asymmetric Cellulose Nanocrystals: Thiolation of Reducing End Groups via NHS-EDC Coupling. Cellulose 2014, 21 (6), 4209–4218. 10.1007/s10570-014-0426-9.

[ref26] Zoppe J. O.; Dupire A. V. M.; Lachat T. G. G.; Lemal P.; Rodriguez-Lorenzo L.; Petri-Fink A.; Weder C.; Klok H.-A. Cellulose Nanocrystals with Tethered Polymer Chains: Chemically Patchy versus Uniform Decoration. ACS Macro Lett. 2017, 6 (9), 892–897. 10.1021/acsmacrolett.7b00383. 35650886

[ref27] Risteen B.; Delepierre G.; Srinivasarao M.; Weder C.; Russo P.; Reichmanis E.; Zoppe J. Thermally Switchable Liquid Crystals Based on Cellulose Nanocrystals with Patchy Polymer Grafts. Small 2018, 14 (46), 1802060 10.1002/smll.201802060. 30198146

[ref28] Lin F.; Cousin F.; Putaux J.-L.; Jean B. Temperature-Controlled Star-Shaped Cellulose Nanocrystal Assemblies Resulting from Asymmetric Polymer Grafting. ACS Macro Lett. 2019, 8 (4), 345–351. 10.1021/acsmacrolett.8b01005. 35651135

[ref29] Chemin M.; Moreau C.; Cathala B.; Villares A. Asymmetric Modification of Cellulose Nanocrystals with PAMAM Dendrimers for the Preparation of PH-Responsive Hairy Surfaces. Carbohydr. Polym. 2020, 249, 116779 10.1016/j.carbpol.2020.116779. 32933703

[ref30] Sipahi-Sağlam E.; Gelbrich M.; Gruber E. Topochemically Modified Cellulose. Cellulose 2003, 10 (3), 237–250. 10.1023/A:1025151701985.

[ref31] Sadeghifar H.; Filpponen I.; Clarke S. P.; Brougham D. F.; Argyropoulos D. S. Production of Cellulose Nanocrystals Using Hydrobromic Acid and Click Reactions on Their Surface. J. Mater. Sci. 2011, 46 (22), 7344–7355. 10.1007/s10853-011-5696-0.

[ref32] Huang J.-L.; Li C.-J.; Gray D. G. Cellulose Nanocrystals Incorporating Fluorescent Methylcoumarin Groups. ACS Sustainable Chem. Eng. 2013, 1 (9), 1160–1164. 10.1021/sc400074e.

[ref33] Karaaslan M. A.; Gao G.; Kadla J. F. Nanocrystalline Cellulose/β-Casein Conjugated Nanoparticles Prepared by Click Chemistry. Cellulose 2013, 20 (6), 2655–2665. 10.1007/s10570-013-0065-6.

[ref34] Lowry T. M. CXXV.—Studies of Dynamic Isomerism. I. The Mutarotation of Glucose. J. Chem. Soc., Trans. 1903, 83 (0), 1314–1323. 10.1039/CT9038301314.

[ref35] Matyjaszewski K. Atom Transfer Radical Polymerization (ATRP): Current Status and Future Perspectives. Macromolecules 2012, 45 (10), 4015–4039. 10.1021/ma3001719.

[ref36] Frka-Petesic B.; Guidetti G.; Kamita G.; Vignolini S. Controlling the Photonic Properties of Cholesteric Cellulose Nanocrystal Films with Magnets. Adv. Mater. 2017, 29 (32), 1701469 10.1002/adma.201701469. 28635143

[ref37] Delepierre G.; Eyley S.; Thielemans W.; Weder C.; Cranston E. D.; Zoppe J. O. Patience Is a Virtue: Self-Assembly and Physico-Chemical Properties of Cellulose Nanocrystal Allomorphs. Nanoscale 2020, 12, 17480–17493. 10.1039/D0NR04491A. 32808640

[ref38] Houga C.; Meins J.-F. L.; Borsali R.; Taton D.; Gnanou Y. Synthesis of ATRP-Induced Dextran-b-Polystyrene Diblock Copolymers and Preliminary Investigation of Their Self-Assembly in Water. Chem. Commun. 2007, (29), 3063–3065. 10.1039/b706248f. 17639142

[ref39] Willker W.; Leibfritz D.; Kerssebaum R.; Bermel W. Gradient Selection in Inverse Heteronuclear Correlation Spectroscopy. Magn. Reson. Chem. 1993, 31 (3), 287–292. 10.1002/mrc.1260310315.

[ref40] Schleucher J.; Schwendinger M.; Sattler M.; Schmidt P.; Schedletzky O.; Glaser S. J.; Sorensen O. W.; Griesinger C. A General Enhancement Scheme in Heteronuclear Multidimensional NMR Employing Pulsed Field Gradients J. Biomol. NMR 1994; Vol. 4.301 306 10.1007/BF00175254 8019138

[ref41] Mestrelab Research S.L. Analytical Chemistry Software Solutions, https://mestrelab.com/ (accessed Feb 18, 2021).

[ref42] Wojdyr M. Fityk: A General-Purpose Peak Fitting Program. J. Appl. Crystallogr. 2010, 43 (5), 1126–1128. 10.1107/S0021889810030499.

[ref43] Beck S.; Méthot M.; Bouchard J. General Procedure for Determining Cellulose Nanocrystal Sulfate Half-Ester Content by Conductometric Titration. Cellulose 2015, 22 (1), 101–116. 10.1007/s10570-014-0513-y.

[ref44] International Organization for Standardization. Nanotechnologies — Standard terms and their definition for cellulose nanomaterial. ISO/TS 20477:2017 https://www.iso.org/cms/render/live/en/sites/isoorg/contents/data/standard/06/81/68153.html (accessed Jun 17, 2020).

[ref45] Yang J. C.; Jablonsky M. J.; Mays J. W. NMR and FT-IR Studies of Sulfonated Styrene-Based Homopolymers and Copolymers. Polymer 2002, 43 (19), 5125–5132. 10.1016/S0032-3861(02)00390-7.

[ref46] Yin Y.; Tian X.; Jiang X.; Wang H.; Gao W. Modification of Cellulose Nanocrystal via SI-ATRP of Styrene and the Mechanism of Its Reinforcement of Polymethylmethacrylate. Carbohydr. Polym. 2016, 142, 206–212. 10.1016/j.carbpol.2016.01.014. 26917392

[ref47] Dong X. M.; Kimura T.; Revol J.-F.; Gray D. G. Effects of Ionic Strength on the Isotropic-Chiral Nematic Phase Transition of Suspensions of Cellulose Crystallites. Langmuir 1996, 12 (8), 2076–2082. 10.1021/la950133b.

[ref48] Revol J.-F.; Bradford H.; Giasson J.; Marchessault R. H.; Gray D. G. Helicoidal Self-Ordering of Cellulose Microfibrils in Aqueous Suspension. Int. J. Biol. Macromol. 1992, 14 (3), 170–172. 10.1016/S0141-8130(05)80008-X. 1390450

[ref49] Tsarevsky N. V.; Matyjaszewski K. Green” Atom Transfer Radical Polymerization: From Process Design to Preparation of Well-Defined Environmentally Friendly Polymeric Materials. Chem. Rev. 2007, 107 (6), 2270–2299. 10.1021/cr050947p. 17530906

[ref50] Hall C. D.; Leeding C. J.; Jones S.; Case-Green S.; Sanderson I.; van Hoorn M. Kinetics and Mechanism of the Formation of Methacrylamide from 2-Methyl-2-Sulphatopropionamide in Strong Acid Media. J. Chem. Soc., Perkin Trans. 2 1991, (3), 417–422. 10.1039/p29910000417.

[ref51] Lai J. T. Hindered Amines. General Synthesis of α-(Tert-Butylamino)-Isobutyramides. Tetrahedron Lett. 1982, 23 (6), 595–598. 10.1016/S0040-4039(00)86899-9.

[ref52] Yin Y.; Berglund L.; Salmén L. Effect of Steam Treatment on the Properties of Wood Cell Walls. Biomacromolecules 2011, 12 (1), 194–202. 10.1021/bm101144m. 21133402

[ref53] Lin K.-H.; Hu D.; Sugimoto T.; Chang F.-C.; Kobayashi M.; Enomae T. An Analysis on the Electrophoretic Mobility of Cellulose Nanocrystals as Thin Cylinders: Relaxation and End Effect. RSC Adv. 2019, 9 (58), 34032–34038. 10.1039/C9RA05156B. PMC9073953 35528898

[ref54] Foster E. J.; Moon R. J.; Agarwal U. P.; Bortner M. J.; Bras J.; Camarero-Espinosa S.; Chan K. J.; Clift M. J. D.; Cranston E. D.; Eichhorn S. J.; Fox D. M.; Hamad W. Y.; Heux L.; Jean B.; Korey M.; Nieh W.; Ong K. J.; Reid M. S.; Renneckar S.; Roberts R.; Shatkin J. A.; Simonsen J.; Stinson-Bagby K.; Wanasekara N.; Youngblood J. Current Characterization Methods for Cellulose Nanomaterials. Chem. Soc. Rev. 2018, 47 (8), 2609–2679. 10.1039/C6CS00895J. 29658545

[ref55] Brinsmead K. H.; Kear R. W. Behavior of Sodium Chloride during the Combustion of Carbon. Fuel (United Kingdom) 1956, 35.

[ref56] Beck S.; Bouchard J.; Berry R. Dispersibility in Water of Dried Nanocrystalline Cellulose. Biomacromolecules 2012, 13 (5), 1486–1494. 10.1021/bm300191k. 22482888

[ref57] Schütz C.; Bruckner J. R.; Honorato-Rios C.; Tosheva Z.; Anyfantakis M.; Lagerwall J. P. F. From Equilibrium Liquid Crystal Formation and Kinetic Arrest to Photonic Bandgap Films Using Suspensions of Cellulose Nanocrystals. Crystals 2020, 10 (3), 199 10.3390/cryst10030199.

[ref58] Dong X.; Revol J.-F.; Gray D. Effect of Microcrystallite Preparation Conditions on the Formation of Colloid Crystals of Cellulose. Cellulose 1998, 5 (1), 19–32. 10.1023/A:1009260511939.

[ref59] Morandi G.; Thielemans W. Synthesis of Cellulose Nanocrystals Bearing Photocleavable Grafts by ATRP. Polym. Chem. 2012, 3 (6), 1402–1407. 10.1039/c2py20069d.

