# Supplementary material for: Challenges in Synthesis and Analysis of Asymmetrically Grafted Cellulose Nanocrystals via Atom Transfer Radical Polymerization
Source: Biomacromolecules. 2021 Jun 1;22(6):2702–17. doi: 10.1021/acs.biomac.1c00392 (PMC8382247; doi:10.1021/acs.biomac.1c00392)
Supplement: Supplementary file 1 — bm1c00392_si_001.pdf [file bm1c00392_si_001.pdf]

Supporting Information

Challenges in Synthesis and Analysis of Asymmetrically Grafted Cellulose Nanocrystals *via* Atom Transfer Radical Polymerization

Gwendoline Delepierre[+], Katja Heise[+], Kiia Malinen, Tetyana Koso, Leena Pitkänen, Emily D. Cranston, Ilkka Kilpeläinen, Mauri A. Kostiainen, Eero Kontturi, Christoph Weder, Justin O. Zoppe, Alistair W.T. King*

G. Delepierre, C. Weder,
Adolphe Merkle Institute, University of Fribourg,
Chemin des Verdiers 4, 1700 Fribourg, Switzerland

K. Heise, K. Malinen, L. Pitkänen, E. Kontturi, M. A. Kostiainen
Department of Bioproducts and Biosystems, Aalto University,
P.O. Box 16300, FI-00076, Aalto, Espoo, Finland

J.O. Zoppe,

Department of Materials Science & Engineering, Universitat Politècnica de Catalunya, Av. Eduard Maristany 10-14, 08019 Barcelona, Spain

E.D. Cranston,

University of British Columbia,

2424 Main Mall, Vancouver, BC V6 T 1Z4, Canada

T. Koso, I. Kilpeläinen, A.W.T. King,
Materials Chemistry Division, Chemistry Department, University of Helsinki,

A.I. Virtasen aukio 1, FI-00560 Helsinki, Finland

Correspondence:

*alistair.king@helsinki.fi

Number of Pages: 20

Number of Figure: 20

Number of Tables: 2

Contents

S1. Characterization of the pristine CNCs ***S3***

S2. Cellobiose model compounds ***S4***

S2a. One-step pathway – model compound M1 *S5*

S2b. Two-step pathway – model compounds M2 and M3 *S7*

S2c. Analytical and preparative scale liquid chromatography *S11*

S3. ATRP-initiator stability in D_2O – NMR experiment ***S12***

S4. NMR methods and remaining NMR data ***S13***

S4a. Diffusion-edited ^1H experiments ***S13***

S4b. Multiplicity-edited HSQC experiments *S14*

S4c. HSQC-TOCSY experiments *S14*

S4d. HMBC experiments *S14*

S4e. Peak-fitting the ^1H CNC-RE-g-PSS spectra for wt% PSS determination *S15*

S4e. Remaining NMR data *S16*

S5. Characterization of the polymer grafted CNCs – CNC-RE-*g*-PSS-1 and CNC-RE-*g*-PSS-2 ***S18***

References ***S19***

S1. Characterization of the pristine CNCs

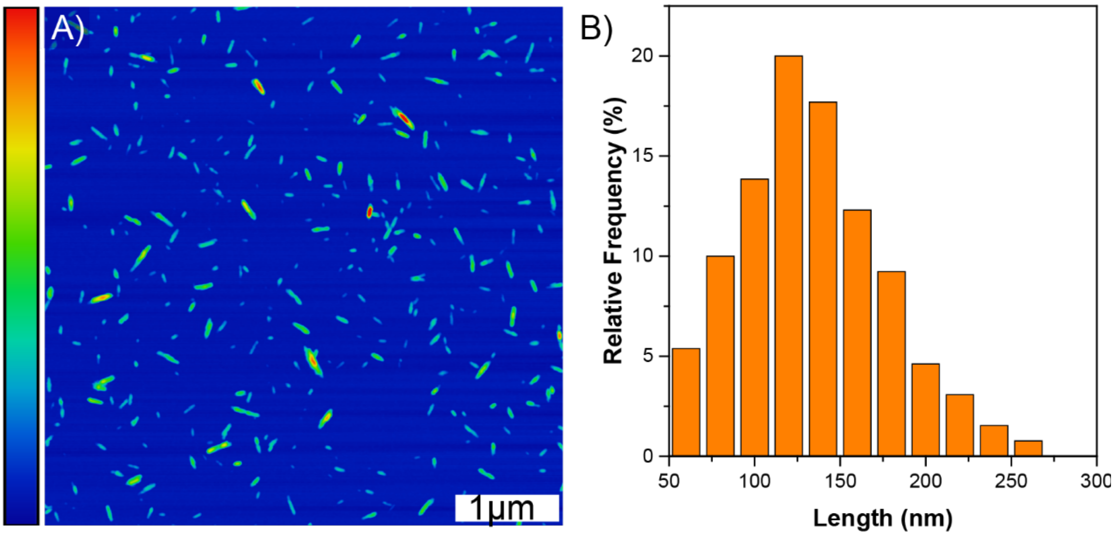

Figure S1. Atomic force microscopy image of neat CNCs on poly-L-lysine coated mica (left). Length distribution histogram of neat CNCs (N=3500), with a length of 133 ± 62 nm, a height of 7 ± 3 nm and an aspect ratio of 21 ± 8.

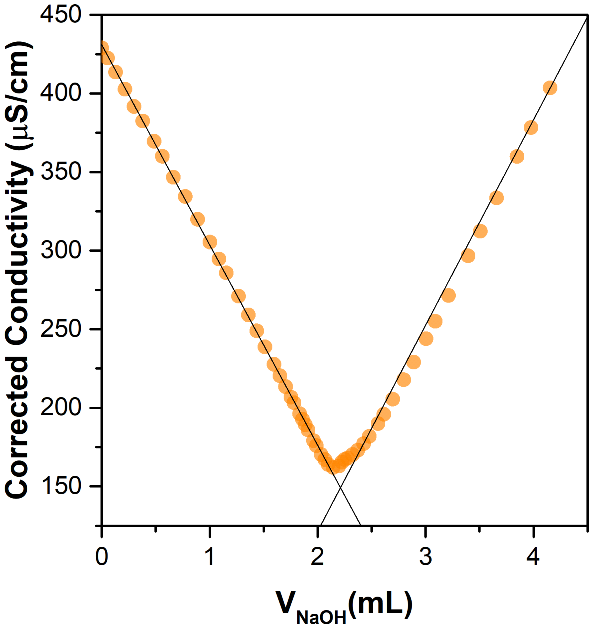

Figure S2. Conductometric titration data used to determine the concentration of sulfate half-ester groups on the surface of the neat CNCs. The CNCs contain 235 ± 3 µmol -OSO_3^-/g CNC.

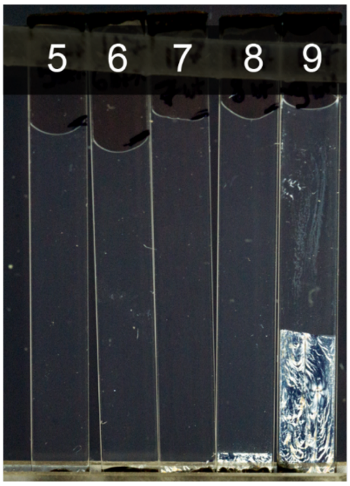

Figure S3. Photograph taken between crossed polarizers of capillaries filled with aqueous suspensions of neat CNCs (the concentrations are indicated in wt% at the top of the capillaries) showing the formation of a chiral nematic phase at a CNC concentration of 8 wt%. All capillaries contain 1 mM NaCl in order to achieve an electrical double layer of 9.7 nm.

S2. Cellobiose model compounds

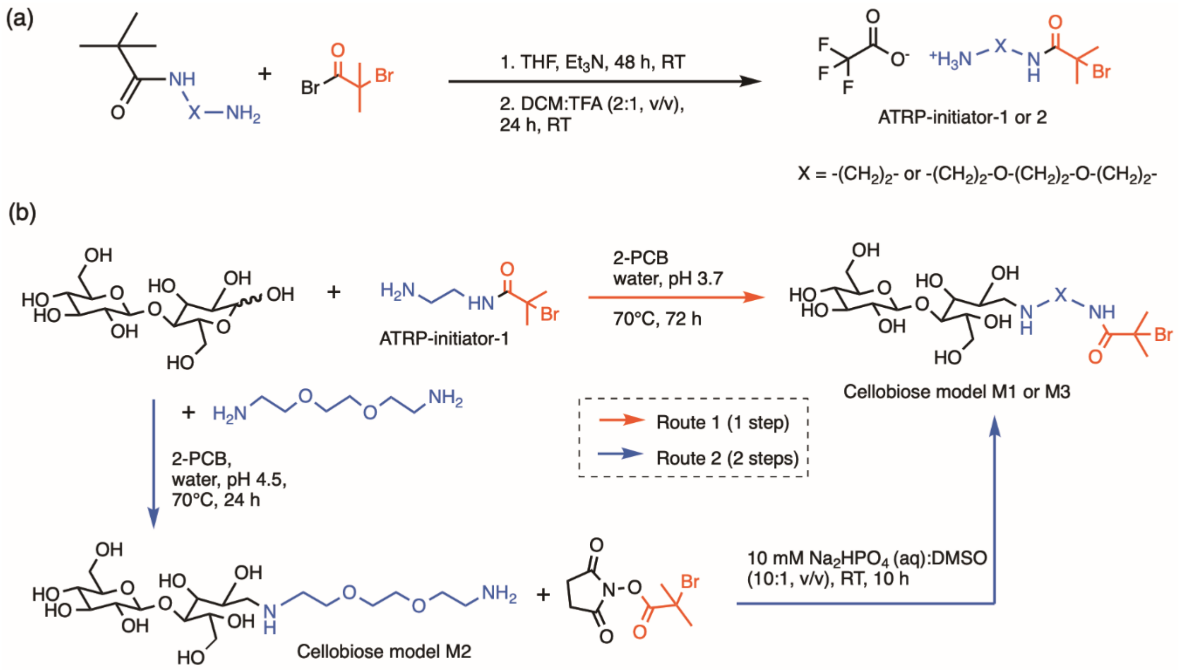

Figure S4. Reaction schemes showing a) the synthesis of the amino-terminated ATRP-initiators-1 and 2, and b) the two reaction routes for the synthesis of the cellobiose model compounds *via* a direct reductive amination (M1) or following a two-step pathway that includes (i) attachment of a diamine *via* reductive amination (M2) and (ii) attachment of an NHS-activated ATRP-initiator (M3). 2-Picoline borane (2-PCB) was used as reductant in both reductive aminations.

S2a. One-step pathway – cellobiose model compound M1

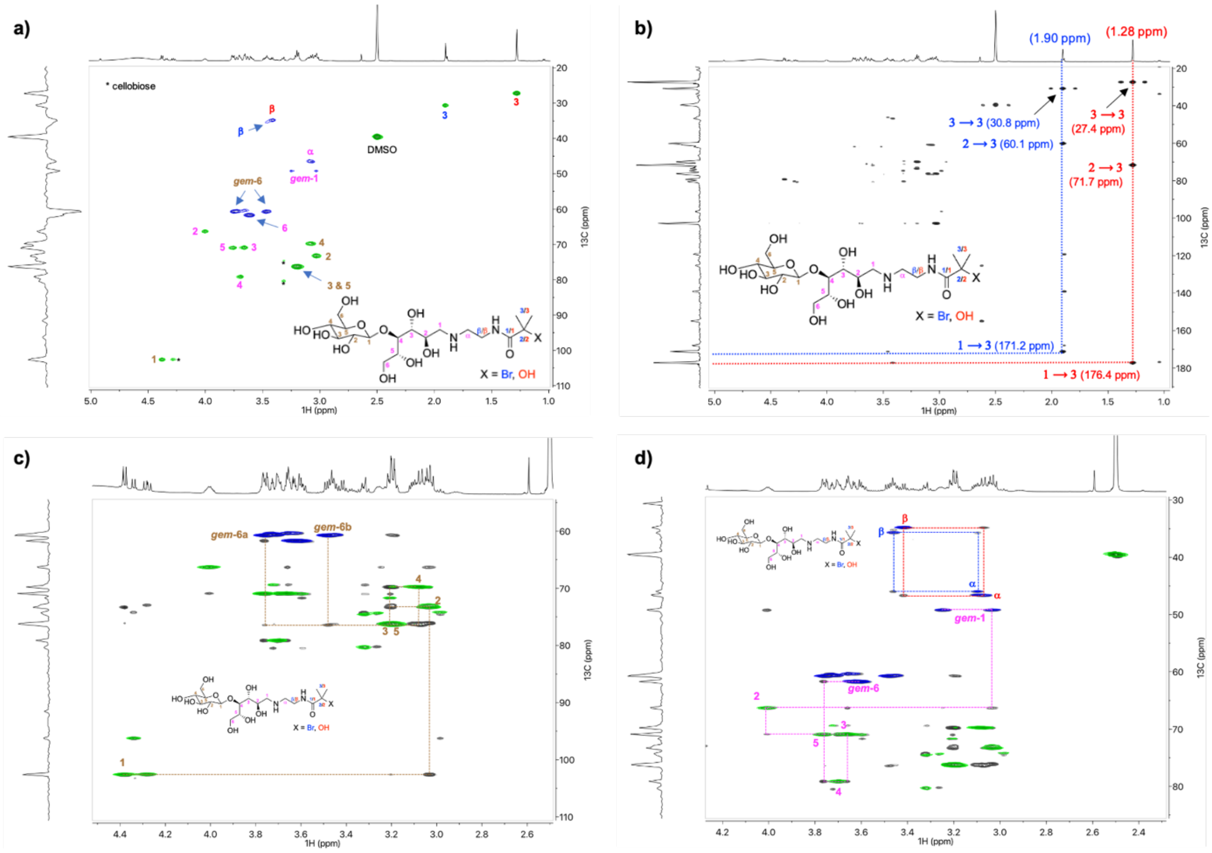

Figure S5. 2D assignments for cellobiose model M1 (in DMSO-d_6 at 65 °C): a) Multiplicity-edited HSQC, b) HMBC, c) HSQC-TOCSY (15 ms mixing time) showing the glucose spin-system (in brown), and d) HSQC-TOCSY (15 ms mixing time) showing the terminal aminated glucose moiety (in pink) and linking amine spin systems (in blue and red).

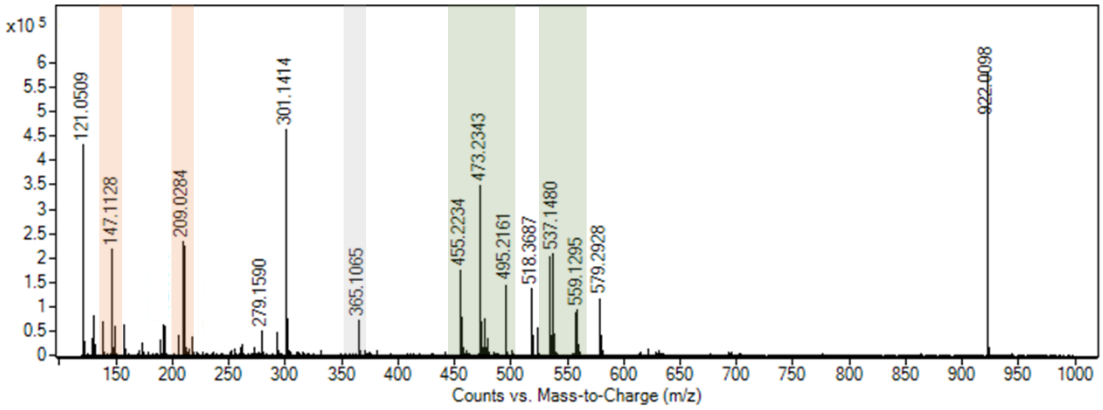

m/z	Assignment
147.11	Unreacted ATRP-initiator-1 (OH) $(M+H)^+$
209.03	Unreacted ATRP-initiator-1 (Br) $(M+H)^+$
365.11	Unreacted cellobiose $(M+Na)^+$
455.22	Model compound M1 (methacryl) $(M+H)^+$
473.23, 495.22	Model compound M1 (OH) $(M+H)^+$, $(M+Na)^+$
537.15, 559.13	Model compound M1 (Br) $(M+H)^+$, $(M+Na)^+$

Figure S6 HRMS spectrum (ESI scan, rt: 0.895 min) of the cellobiose model compound M1 ($C_{18}H_{35}BrN_2O_{11}$) and the corresponding signal assignments revealing side reactions on the ATRP-initiator residue and showing that a full conversion of cellobiose with ATRP-initiator-1 was not achieved.

S2b. Two-step pathway – cellobiose model compounds M2 and M3

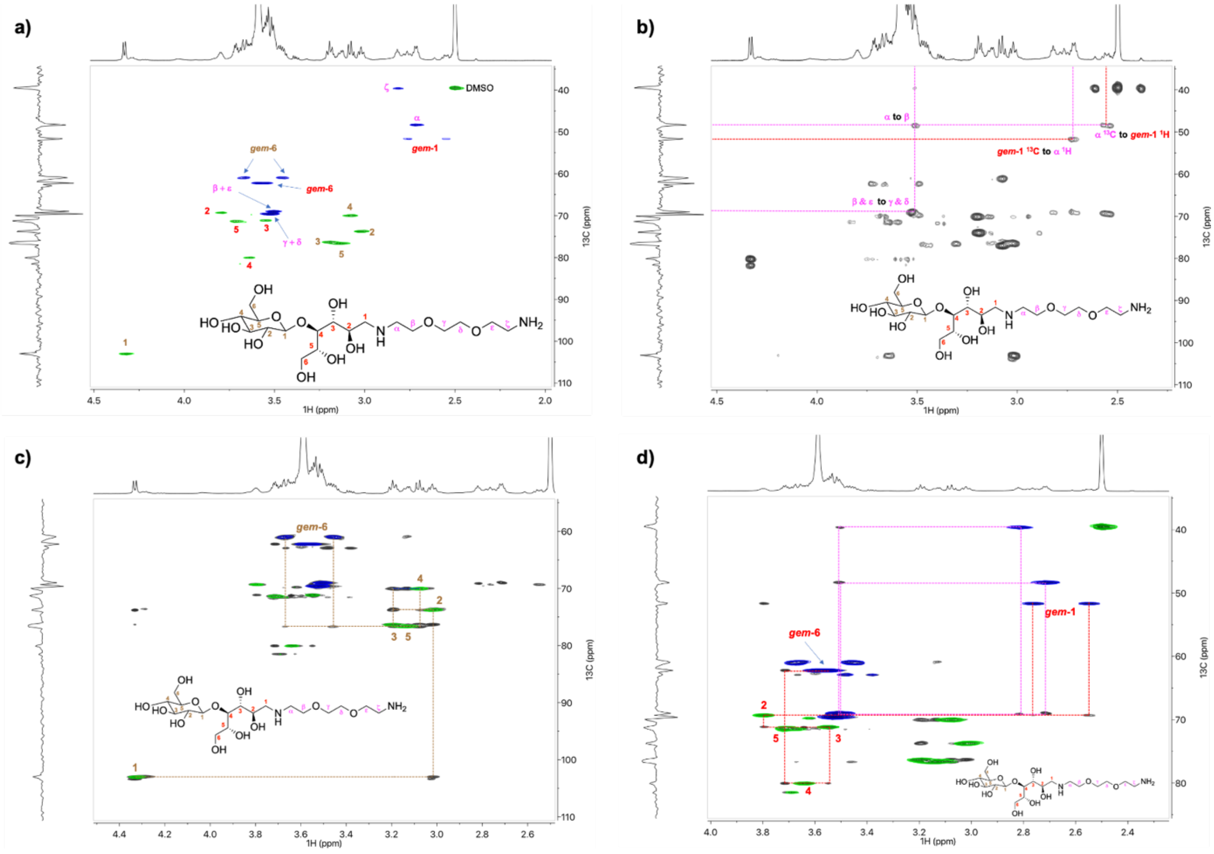

Figure S7. 2D assignments for cellobiose model M2 (in DMSO-d_6 at 65 °C): a) Multiplicity-edited HSQC, b) HMBC, c) HSQC-TOCSY (15 ms mixing time) showing the glucose spin-system (in brown), and d) HSQC-TOCSY (15 ms mixing time) showing the terminal aminated glucose moiety (in pink) and amine spin systems (in blue and red).

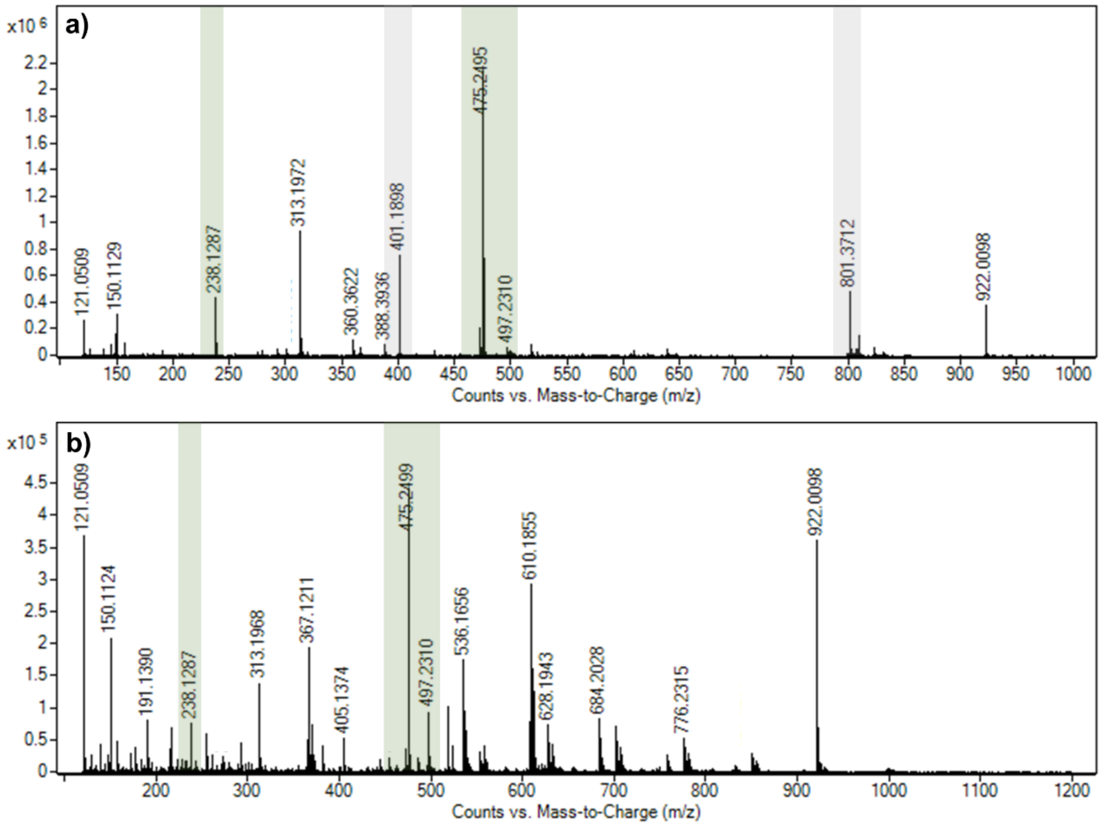

m/z	Assignment
238.13	Model compound M2 $(M+2H)^{+2}$
475.25, 497.23	Model compound M2 $(M+H)^{+}$, $(M+Na)^{+}$
401.19, 801.37	Crosslinked cellobiosylamine $(M+H)^{+}$, $(M+2H)^{+2}$

Figure S8. HRMS spectra (ESI scans – a) rt: 0.900 min, b) rt: 0.927 min) and the corresponding signal assignments of the cellobiose model compound M2 ($C_{18}H_{38}BrN_2O_{12}$): a) "crude" compound before and b) "purified" compound (fraction 1) after purification by preparative-scale HPLC. The spectrum of the "crude" compound (a) shows partial end-to-end attachment of two cellobiose molecules by the diamine (m/z = 801.37, $(M+H)^{+}$).

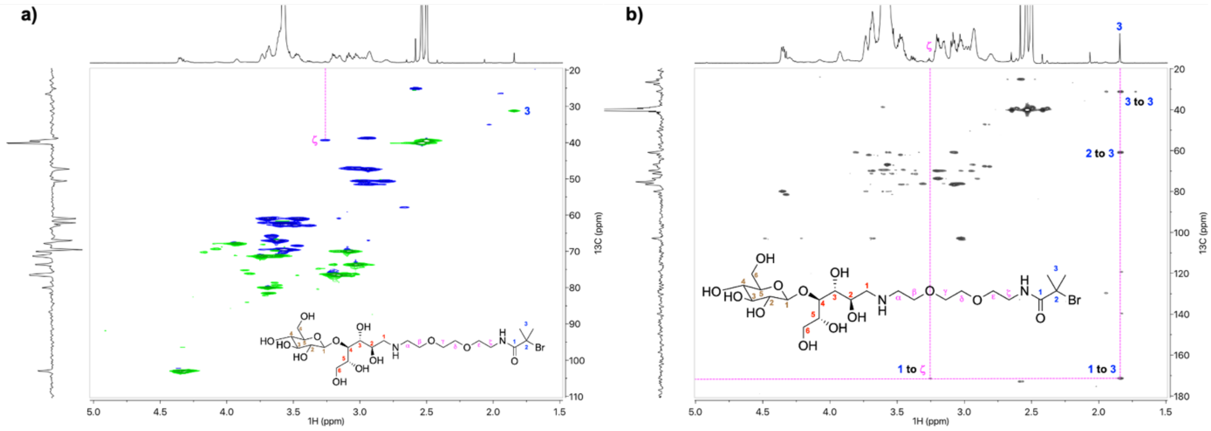

Figure S9 2D spectra for cellobiose model M3 (in DMSO-d_6 at 65 °C): a) Multiplicity-edited HSQC, b) HMBC; The highlighted correlations shown are for the M3 ζ to BiBB-Br spin system. The full spectrum is not assigned as this is a mixture of M2 and M3, with M3 present only in a small amount.

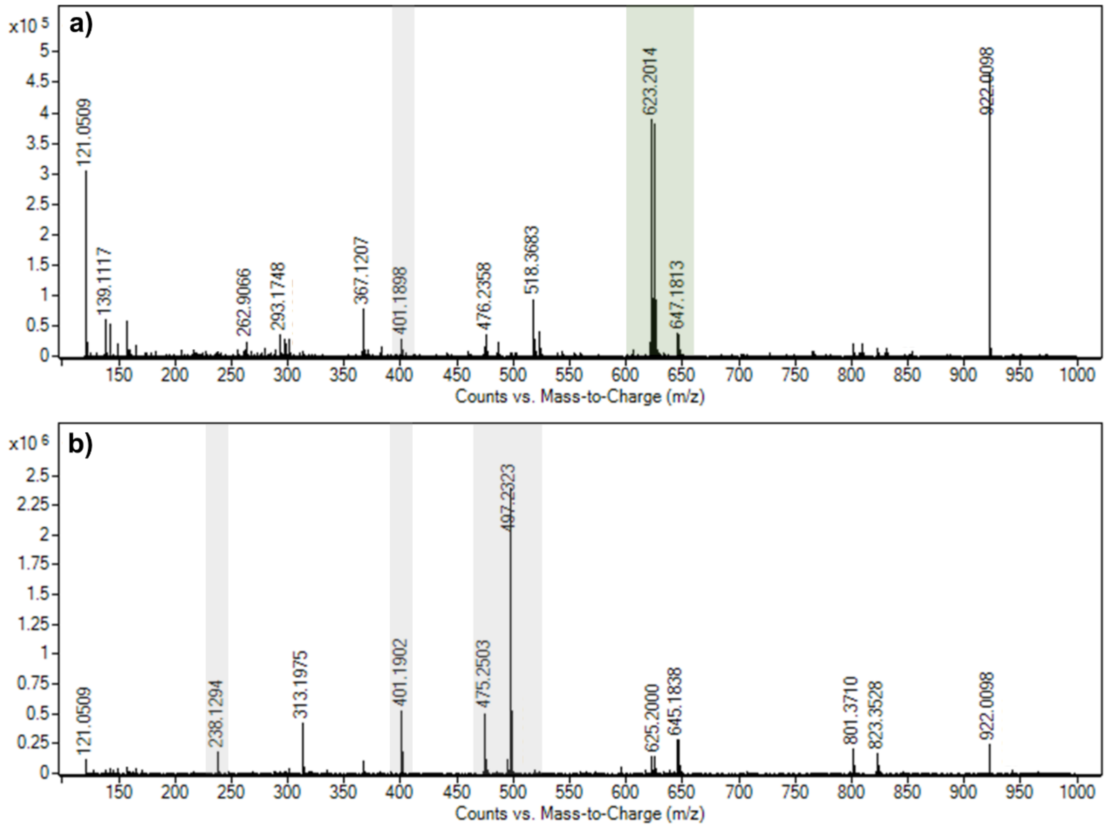

**m/z** (spectrum)	**Assignment**
238.13, 475.25, 497.23 (b)	Unreacted model compound M2 $(M+2H)^{2+}$, $(M+H)^{+}$, $(M+Na)^{+}$
401.19 (a/b)	Crosslinked cellobiosylamine $(M+2H)^{+2}$
623.20, 647.18 (a)	Model compound M3 $(M+H)^{+}$, $(M+Na)^{+}$

Figure S10. HRMS spectra (ESI scans – a) rt: 0.892 min, b) rt: 0.955 min) and the corresponding signal assignments of the cellobiose model compound M3 ($C_{22}H_{43}BrN_2O_{13}$). a) ESI scan of the product showing a clear signal for M3. b) Ionization assisted by addition of formic acid shows high amounts of unreacted cellobiosylamine (model M2) and end-to-end attached (cross-linked) cellobiose in the product revealing that the conversion of M2 to M3 was not complete.

S2c. Analytical and semi-preparative scale liquid chromatography

Liquid chromatographic (LC) separations for the purification of the cellobiosylamine (model compound M2) were started by a method development using an analytical scale column. The developed method was then scaled for semi-preparative LC. The analytical HPLC instrument consisted of Agilent 1260 system including a binary pump, autosampler, column oven, and an evaporative light scattering detector (Agilent 1290 ELSD). A Phenomenex Luna® Omega 3 µm PSC18 (100 × 2.1 mm) column was used for the separation. The column was thermoregulated to 40 °C and ELSD nebulizer and evaporation temperatures were 60 °C and 80 °C, respectively. The flow rate was 0.4 ml/min. The sample was dissolved in DI water (1 mg/mL) and the injection volume was 20 µL. Since the refractive index detector was used in semi-preparative LC, the aim was to develop an isocratic LC method. Different ACN/water ratios were tested (Figure S13), and an ACN/water ratio of 70/30 (v/v) seemed optimal for separation.

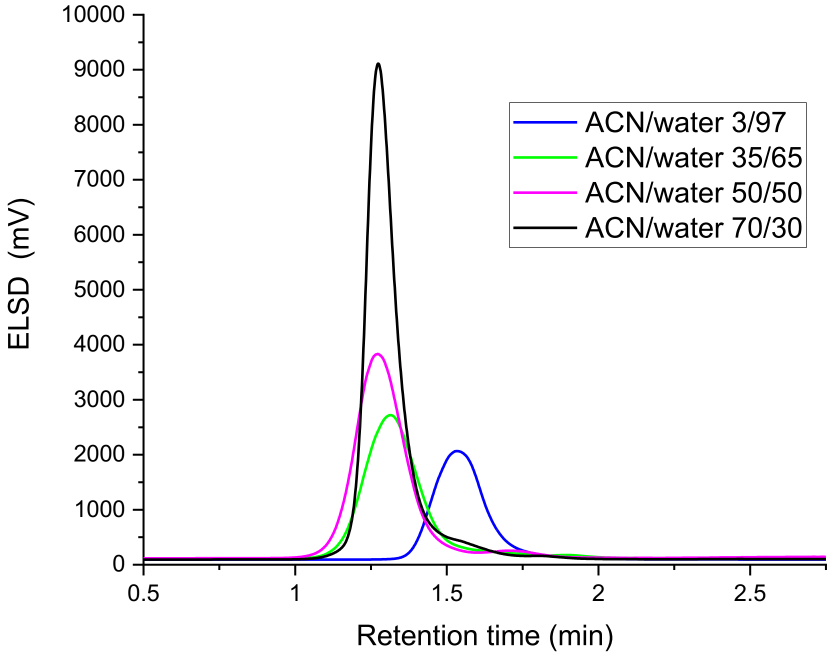

Figure S11. HPLC chromatogram (evaporative light scattering detector) of the “crude” (before purification by preparative-scale HPLC) cellobiose model compound M2 using different mobile phase compositions with varied ratios (v/v) between acetonitrile and water.

Semi-preparative separation (Figure S14) was accomplished using a Shimadzu preparative LC consisting of two LC-20AP pumps, SIL-10AP autosampler, RID-20A refractive index detector,

and FRC-10A fraction collector. A semi-preparative Luna® Omega 5 µm PSC18 (250 × 10 mm) column from Phenomenex was used for the separation. ACN/water (70/30, v/v) was used as eluent with a flow rate of 3 mL/min. The injection volume was 50 or 100 µL. Two fractions were collected from 58 injections (fraction 1: 3.80-4.30 min, fraction 2: 4.30-4.65).

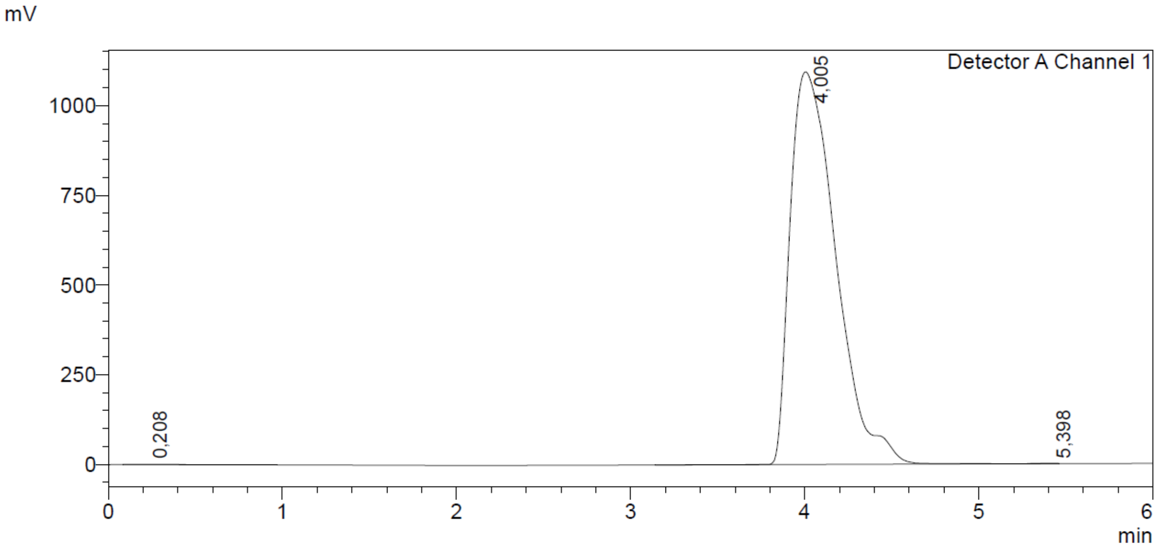

Figure S12. Chromatogram of the "crude" (before purification) cellobiose model compound M2 acquired during semi-preparative-scale HPLC (RI detection) using acetonitrile:water = 70:30 (v/v) as mobile phase.

S3. ATRP-initiator stability in D_2O – NMR experiment

For the NMR experiment (Figure S13), 10 mg of ATRP-initiator-1 were dissolved in 0.7 mL D_2O (Eurisotop®, D215H, 99.96 % D) at room temperature and then transferred to 5-mm Aldrich® ColorSpec® NMR tubes (Sigma-Aldrich). For the heating experiment, ATRP-initiator-1, dissolved in D_2O, was directly heated in the NMR tube for 24 h at 70 °C. All NMR spectra were recorded on a 400-MHz Avance III spectrometer (Bruker) at 25 °C. The AVIII400 is equipped with a 5-mm liquid-state probe. For standard ^{1}H 1D NMR spectra ('zg30' in the Bruker TopSpin 4.0 pulse program library), 8 transient scans (ns) were collected. For qualitative ^{13}C 1D NMR spectra (pulse program 'zgpg30'), 256 transient scans were collected. Multiplicity-edited Heteronuclear Single Quantum Correlation (HSQC) experiments in echo/antiecho acquisition mode ('hsqcedetgpsisp2.3' in the Bruker TopSpin 4.0 pulse program library) were recorded with 512 increments (TD) in f1. Sweep-widths (SW) were 13.0 and 165 ppm, with transmitter offsets (O1P) of 6.0 and 80 ppm, respectively. 16 dummy scans (DS) and 4 transient scans (NS) were collected with an acquisition time (aq) of 0.099 s for f2 and a

relaxation delay (D1) of 1.5 s. Heteronuclear multiple bond correlation (HMBC) experiments ('hmbcgpl3nd' in the Bruker TopSpin 4.0 pulse program library) were recorded with 512 increments in f1. Sweep-widths were 13.0 and 235 ppm, with a transmitter frequency offset of 6.0 and 110 ppm. There were 16 dummy scans, 4 transient scans, an acquisition time of 0.19 s for f2 and a relaxation delay of 1.5 s. The CH coupling value (cnst2) for the low-pass filter was 145 Hz and the long-range CCH coupling value (cnst13) was 8 Hz. All spectra were processed using Bruker TopSpin 4.0.9 and/or MestReNova 14.2.0 software.

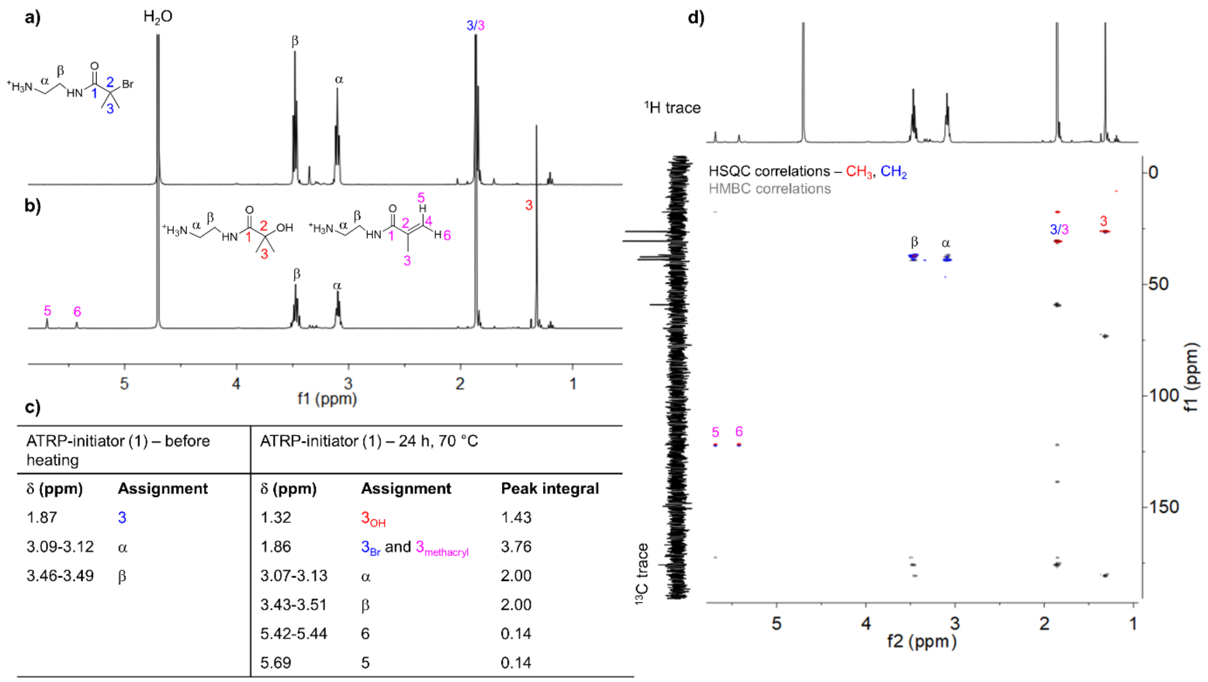

ATRP-initiator (1) – before heating		ATRP-initiator (1) – 24 h, 70 °C		
δ (ppm)	Assignment	δ (ppm)	Assignment	Peak integral
1.87	3	1.32	3_{OH}	1.43
3.09-3.12	α	1.86	3_{Br} and $3_{methacryl}$	3.76
3.46-3.49	β	3.07-3.13	α	2.00
		3.43-3.51	β	2.00
		5.42-5.44	6	0.14
		5.69	5	0.14

Figure S13. NMR analysis of ATRP initiator-1. a) ^1H spectrum just after dissolution of initiator-1 in D_2O (with residual water) and b) after heating for 24 h at 70 °C. c) The corresponding signal assignments and peak integrals for spectrum b) which serve as an estimate for the conversion of BiBB-Br to BiBB-OH. d) ^1H-^{13}C multiplicity-edited HSQC spectrum of initiator 1 after heating in D_2O showing overlayed HMBC-correlations.

S4. NMR methods and remaining NMR data

S4a. Diffusion-edited ^1H experiments

The diffusion-edited ^1H experiment used a 1D bipolar-pulse pair with stimulated echo (BPPSTE),[1] diffusion-ordered spectroscopy (DOSY) pulse sequence (Bruker pulse program 'ledbpgp2s1d'), with 1 s relaxation delay (d1), 0.5 s acquisition time (aq), 16 dummy scans (ds) and 512 transient scans (ns), a sweep-width (sw) of 20 ppm with the transmitter offset on 6.1 ppm (o1p), a diffusion time (d20) of 200

ms, a gradient recovery delay (d16) of 0.2 ms, an eddy current delay (d21) of 5 ms, a diffusion gradient pulse duration (p30) of 2.5 ms and a z-gradient strength (gpz6) of 90% at $\geq$ 50 G/cm (probe z-gradient strength). Shorter and longer runs were performed by running multiples of 16 only for the number of transient scans (ns).

S4b. Multiplicity-edited HSQC experiments

The HSQC experiments used a sensitivity-improved multiplicity-edited phase sensitive HSQC sequence, with echo/antiecho-TPPI gradient selection and adiabatic pulses (Bruker pulse program 'hsqcedetgpsisp2.2').[2–5] Typical parameters are as follows: spectral widths (sw) were 13.03 and 165 ppm, with transmitter offsets (o1p) of 6.18 and 75 ppm, for ^{1}H and ^{13}C dimensions, respectively. The time-domain size (td1) in the indirectly detected ^{13}C-dimension (f1) was typically 512 or 1024, corresponding to 256 or 512 t_1-increments for the real spectrum. There were 16 dummy scans (ds), typically 8 (or multiples of 8) scans (ns), an acquisition time (aq) of 0.065 s for f2 and a relaxation delay of 1.5 s. Window functions were typically sine squared (90 °) in f1 and f2.

S4c. HSQC-TOCSY Experiments

The HSQC-TOCSY experiments used a phase-sensitive HSQC-TOCSY pulse program with the DIPSI-2 isotropic mixing sequence and echo/antiecho-TPPI gradient selection (Bruker pulse program 'hsqcdietgpsisp.2').[2] Typical parameters are as follows: spectral widths (sw) were 13.0 and 200 ppm, with transmitter offsets (o1p) of 6.18 and 90 ppm for ^{1}H and ^{13}C dimensions, respectively. The time-domain size (td1) was 512 or 1024 in the indirectly detected ^{13}C-dimension (f1) dimension. There were 16 dummy scans (ds), typically 8 (or multiples of 8) scans (ns), an acquisition time (aq) of 0.107 s for f2 and a relaxation delay of 1.5 s. The TOCSY mixing delay (d9) was 0.015 s to yield a short-range (COSY-like) TOCSY experiment or 0.12 s to yield a long-range TOCSY experiment, where the full spin-system was typically observed. The latter experiment required typically 2-4 times the scans (ns) to get similar signal-to-noise as the short-range experiment. Window functions were typically sine squared (90 °) in f1 and f2.

S4d. HMBC experiments

The HMBC experiments used a magnitude-mode gradient-enhanced HMBC sequence using a low-pass *J*-filter[6] (Bruker pulse program 'hmbcgplpndqf'). Spectral widths (sw) were 13.0 and 250 ppm, with transmitter offsets (o1p) of 6.3 and 100 ppm for ^{1}H and ^{13}C dimensions, respectively. The time-domain size (td1) in the indirectly detected ^{13}C-dimension (f1) was typically 512. For magnitude mode HMBC

this directly corresponds to 512 t_1-increments for the real spectrum. There were 16 dummy scans (ds), typically 32 scans (ns), an acquisition time (aq) of 0.131 s for f2 and a relaxation delay of 1.5 s. A $^1J_{CH}$ coupling constant value (cnst2) of 145 Hz was used, for setting up the low-pass filter. The polarization transfer delay was optimized for a $^nJ_{CH}$ long-range coupling constant value of 10 Hz (cnst13). Window functions were typically sine bell (0 °) in f1 and f2.

S4e. Peak-fitting the ^{1}H CNC-RE-g-PSS spectra for wt% PSS determination

Wt% PSS in the CNC-RE-*g*-PSS samples was determined from the quantitative ^{1}H spectra (relaxation delay, d1 = 10 s). The procedure is as follows, with a graphical example shown in Figure S14: the 1H spectra were opened in MestreNova; they were phased and baseline corrected (3rd order polynomial); The full spectral regions were saved as 'NMR CSV' files, which are actually tab-separated data, not comma-separated; the file endings were changed to .xy, to allow for opening in the *fityk*[7] peak-fitting software; spline baseline correction was then used, surrounding the cellulose H1-6 and PSS aromatic signals; unwanted areas were deselected for fitting; Gaussian peak guesses were applied to account for the peak volumes, until the residual error was low enough; fitting of the peaks using the available algorithms was not necessary but is best applied after sufficient peaks are guessed – the main issue is to minimise the residual baseline error, which is obvious from the absence of any major residual dispersion. The extracted peak volumes then give the molar quantities of the protons attached to the cellulose backbone vs PSS aromatics.

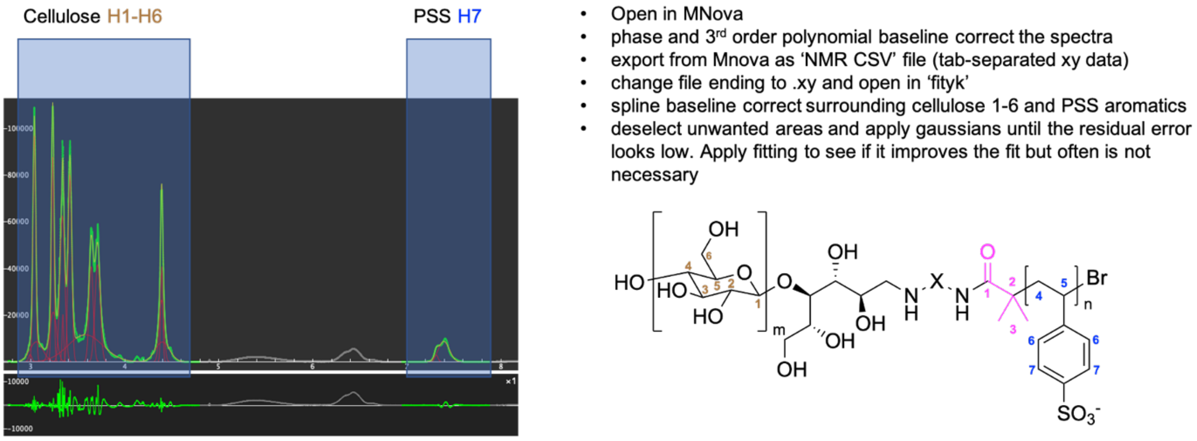

Figure S14. An example of peak-fitting of the CNC-RE-*g*-PSS samples in *fityk*[7].

S4f. Remaining NMR data

The ATRP initiator was assigned using a combination of HSQC & HMBC, to highlight the relevant peaks for the study (Main text, Figure 1). The assignments are given in Figure S15. The assignments for the methacrylamide degradation are given in Figure S16.

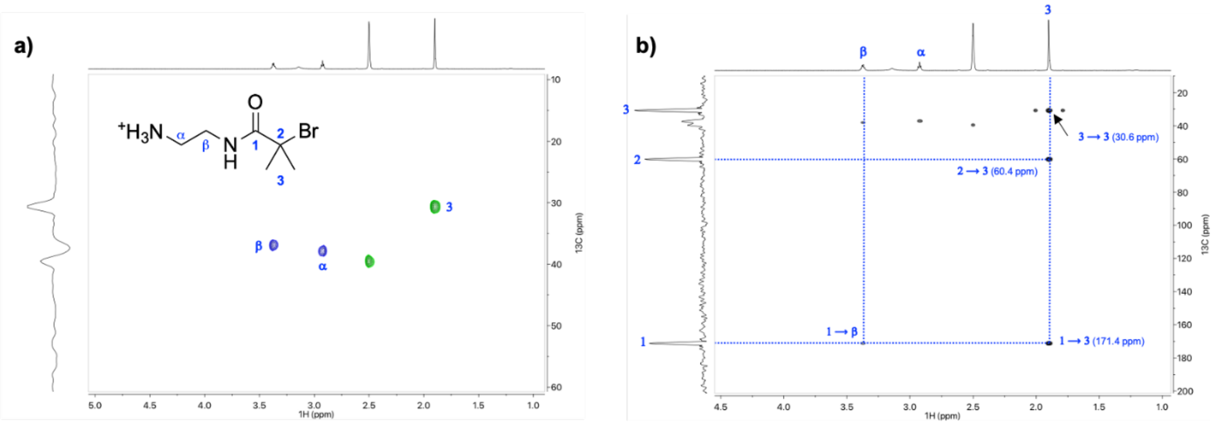

Figure S15. Assigned spectra (in DMSO-d_6 at 65 °C) for the ATRP initiator: a) multiplicity-edited HSQC, and b) HMBC.

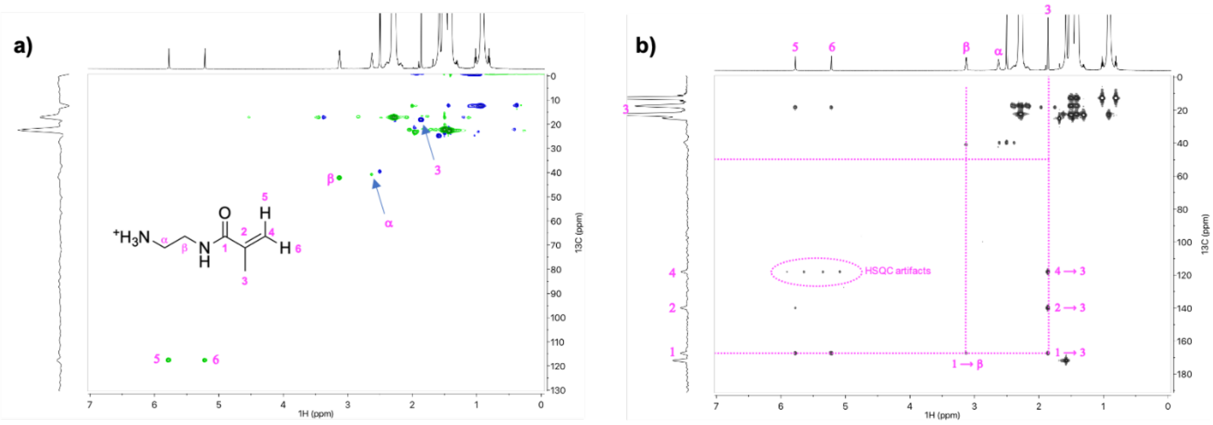

Figure S16. Assigned spectra (in $[P_{4444}][OAc]$:DMSO-d_6 at 65 °C) for the ATRP initiator methacrylamide degradation product: a) multiplicity-edited HSQC, and b) HMBC.

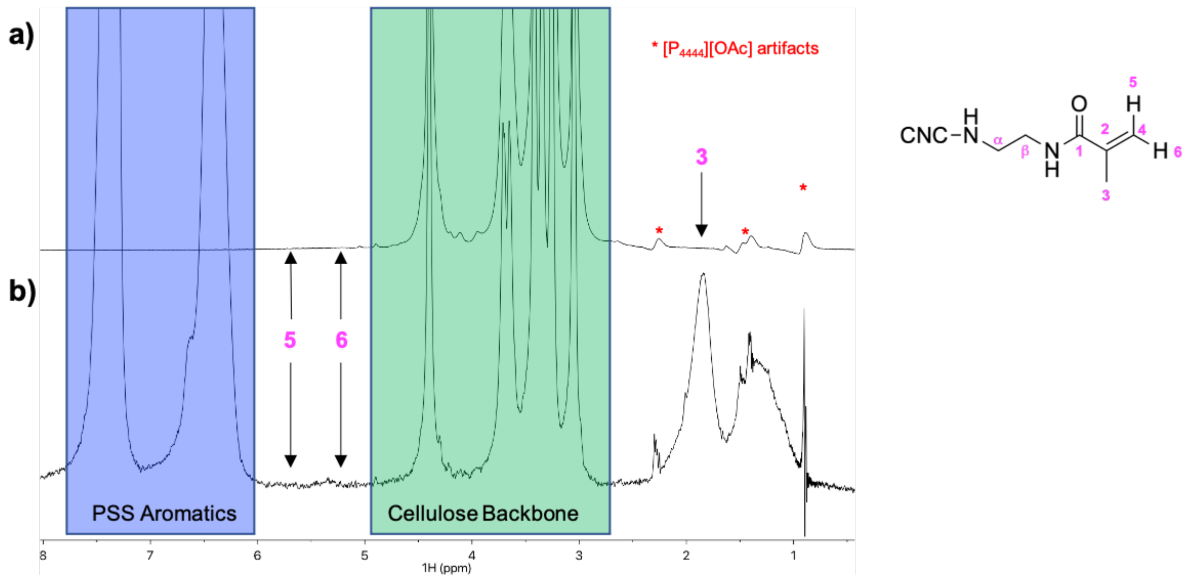

Figure S17. Normalized diffusion-edited ^{1}H spectra (in [P_{4444}][OAc]:DMSO-d_6 at 65 ºC) for a) CNC-RE-*g*-BiBB-2 b) CNC-RE-*g*-PSS-2. Methacrylamide signals are missing from both spectra.

S5. Characterization of the polymer grafted CNCs – CNC-RE-*g*-PSS-1 and CNC-RE-*g*-PSS-2

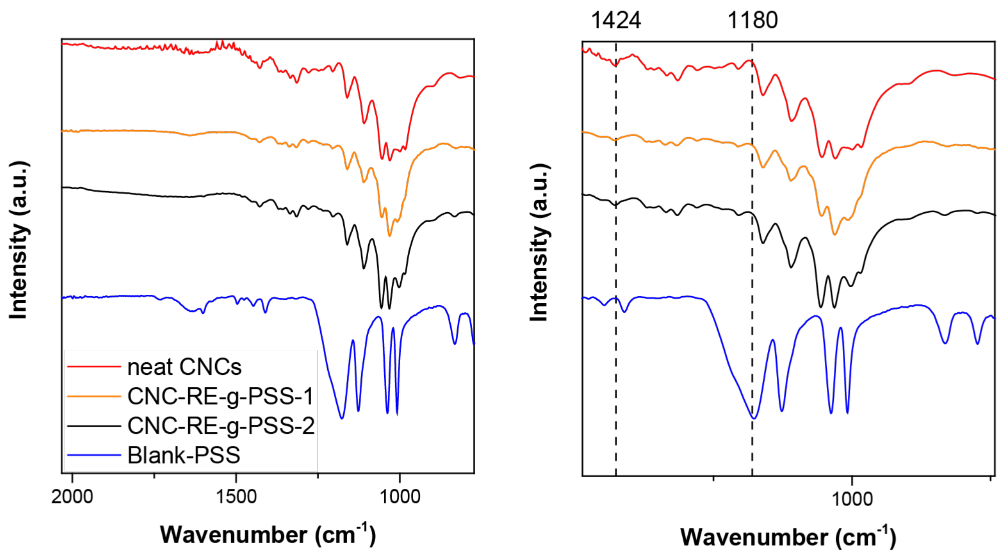

Figure S18. Left) FTIR spectra of the pristine CNCs, CNCs grafted with PSS at their reducing ends, and the PSS homopolymer. Right) Zoomed in spectra to show the bands used for the ratio of PSS and CNCs.

Table S1. DLS and ζ-potential of pristine CNCs and the CNCs grafted with PSS at their reducing ends

	**Apparent size (nm)**	**ζ-potential (mV)**
**Pristine CNCs**	62 ± 0.2	-34 ± 1
**CNC-RE-*g*-BiBB-1**	62 ± 0.3	-32 ± 1
**CNC-RE-*g*-PSS-1**	123 ± 0.2	-41 ± 1
**CNC-RE-*g*-NH_2**	95 ± 0.7	-36 ± 2
**CNC-RE-*g*-BiBB-2**	96 ± 1.2	-32 ± 2
**CNC-RE-*g*-PSS-2**	126 ± 0.3	-37 ± 2

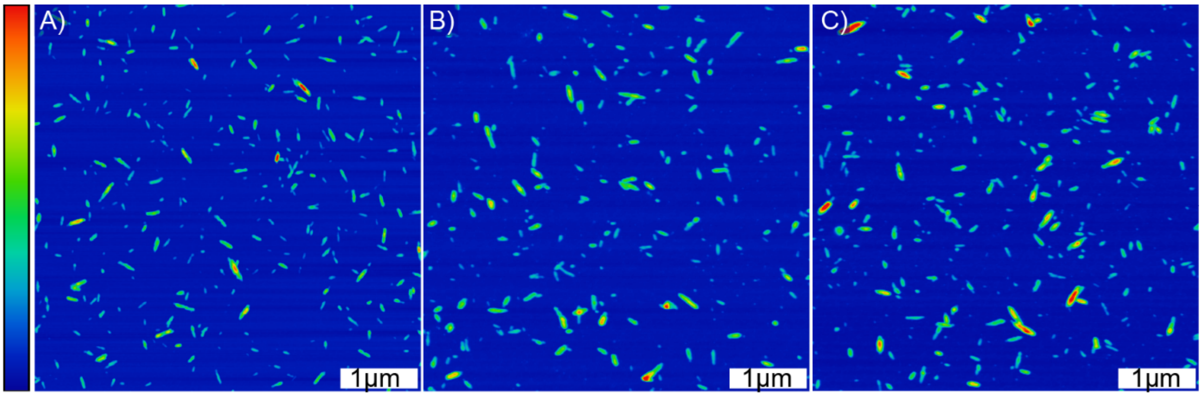

Figure S19. Atomic force microscopy image showing that the rod-like morphology of the CNCs is preserved after the modification.

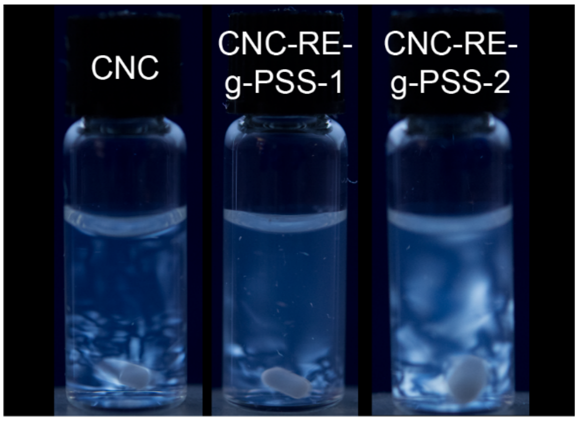

Figure S20. Photograph taken between crossed polarizers of vials containing 0.5 wt% aqueous suspensions of neat CNCs, CNC-RE-*g*-PSS-1**,** and CNC-RE-*g*-PSS-2 (all without salt), showing shear birefringence.

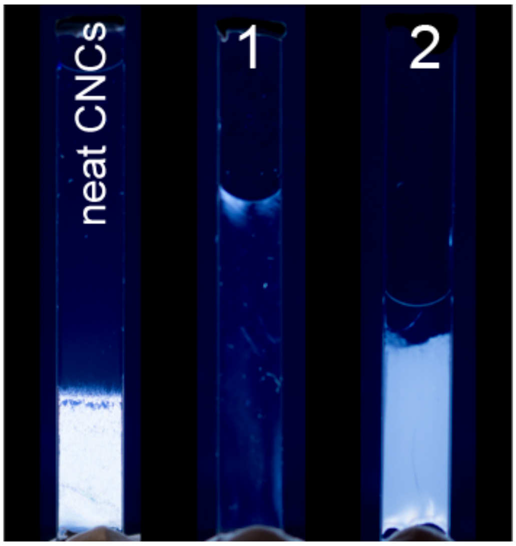

Figure S21. Photograph taken between crossed polarizers of capillaries filled with aqueous suspensions of neat CNCs (9 wt%, 1 mM NaCl), CNC-RE-*g*-PSS-1(4 wt%, 1 mM NaCl), and CNC-RE-*g*-PSS-2 (5 wt%, 1 mM NaCl). The concentrations inside the capillaries vary, as the end-tethered CNCs could not be concentrated up above 4 and 6 wt% for CNC-RE-*g*-PSS-1 and CNC-RE-*g*-PSS-2**,** respectively.

References

(1) Wu, D. H.; Chen, A. D.; Johnson, C. S. An Improved Diffusion-Ordered Spectroscopy Experiment Incorporating Bipolar-Gradient Pulses. *J. Magn. Reson., Series A* **1995**, *115* (2), 260–264. https://doi.org/10.1006/jmra.1995.1176.

(2) Willker, W.; Leibfritz, D.; Kerssebaum, R.; Bermel, W. Gradient Selection in Inverse Heteronuclear Correlation Spectroscopy. *Magn. Reson. Chem.* **1993**, *31* (3), 287–292. https://doi.org/10.1002/mrc.1260310315.

(3) Palmer, A. G.; Cavanagh, J.; Wright, P. E.; Rance, M. Sensitivity Improvement in Proton-Detected Two-Dimensional Heteronuclear Correlation NMR Spectroscopy. *J. Magn. Reson.* **1991**, *93* (1), 151–170. https://doi.org/10.1016/0022-2364(91)90036-S.

(4) Kay, L.; Keifer, P.; Saarinen, T. Pure Absorption Gradient Enhanced Heteronuclear Single Quantum Correlation Spectroscopy with Improved Sensitivity. *J. Am. Chem. Soc.* **1992**, *114* (26), 10663–10665. https://doi.org/10.1021/ja00052a088.

(5) Schleucher, J.; Schwendinger, M.; Sattler, M.; Schmidt, P.; Schedletzky, O.; Glaser, S. J.; Sørensen, O. W.; Griesinger, C. A General Enhancement Scheme in Heteronuclear Multidimensional NMR Employing Pulsed Field Gradients. *J. Biomol. NMR* **1994**, *4* (2), 301–306. https://doi.org/10.1007/BF00175254.

(6) Bax, Ad.; Summers, M. F. Proton and Carbon-13 Assignments from Sensitivity-Enhanced Detection of Heteronuclear Multiple-Bond Connectivity by 2D Multiple Quantum NMR. *J. Am. Chem. Soc.* **1986**, *108* (8), 2093–2094. https://doi.org/10.1021/ja00268a061.

(7). Wojdyr, M. *Fityk*: A General-Purpose Peak Fitting Program. *J. Appl. Crystallogr.* **2010**, *43* (5), 1126–1128. https://doi.org/10.1107/S0021889810030499
